# Effects of steeping duration and concentration of metabolites from rhizosphere bacteria on germinability of cowpea ( *Vigna* *unguiculata*), soybean ( *Glycine max*), sesame ( *Sesamum* *indicum*) and okra ( *Abelmoschus* *esculentus*)

**DOI:** 10.12688/f1000research.137322.1

**Published:** 2023-07-05

**Authors:** Oghenerobor Akpor, Ayotunde Ajinde, Tolulope Ogunnusi

**Affiliations:** 1 Department of Biological Sciences, Afe Babalola University, Ado Ekiti, Ekiti, 360102, Nigeria

**Keywords:** Germinability, rhizobacteria, germinability, seed vigor

## Abstract

Vigorous germination and growth are linked to crop yield. This study was carried out to assess the effects of steeping duration and metabolite concentration on priming of 5 different crops, using the metabolites of five (5) bacterial isolates that were also characterized through Gas Chromatography-Mass Spectrometry (GC-MS). The crop seeds were steeped in cold-extracted metabolites of the 5 isolates for a known period (1, 2, 3, 4, and 5 h) and then also in different metabolites concentrations for a known duration determined as optimal in the first experiment. Characterization of cold-extracted metabolites was also carried out using GCMS. The results of this study revealed that steeping cowpea and soybean for longer durations (< 3 h) could be inhibitory to growth and development. For concentration, it was either a case of lower concentration being optimal or there was no detectable pattern with concentration. The metabolites of the different isolates revealed the present of some common molecules, and some of the GCMS-identified metabolites (e.g., Hexadecanoic acid) have been shown to possess growth promotion properties in other studies. This study highlights that large endosperm seeds such as cowpea and soybean are more prone to the negative effects of steeping for longer durations, and further experiments should be carried out to isolate and purify the bioactive moieties for further studies and onward application.

## Introduction

Seeds are critical inputs in agricultural production. However, poor crop stand, a major production constraint in the developing world ^1^ can result from low-quality seeds and unfavorable edaphic and abiotic factors. This indicates that effort must be made to ensure that seeds are properly prepared, so that their germination and growth and crop yield are not negatively impacted. To this end, seed priming has been proposed and used. The beneficial effects of seed priming on a variety of crops have been confirmed. ^2^^–^^5^ Although, priming, as a plant growth enhancement technique, can be applied at numerous stages in the developmental cycle of a plant, it is typically utilized on seeds for reasons of practicality and simplicity.

Priming usually involves soaking seeds in a solution to kickstart various pre-germinative activities, ^6^^,^^7^ and it typically involves re-drying the seed before plant. ^8^ Several methods of priming that have been shown to enhance the agro-morphic parameters of various crops exist. Hydropriming involves steeping the seeds in water ^9^; osmopriming involves the use of an osmoticum ^10^; halopriming involves soaking in salt solutions ^11^; solid-matrix priming involves priming on a solid material ^12^; and hormonal priming, which involves the use of plant growth regulators such as abscisic acid (ABA), ^13^^,^^14^ gibberellic acids (GAs), ^6^ or salicylic acid (SA). ^15^^–^^17^ Biopriming involving the use of microbial products is a relatively new priming strategy that comes with the advantage of environmental friendliness and may be also be less expensive than most of the priming methods available.

Seed priming has been shown to enhance germination, reduce germination time, and improve seedling vigor. ^18^ Priming also increase the resistance of seeds to environmental stress. ^19^ Positive priming effects have been reported for many crops. ^20^^–^^25^

Although, the exact mechanism of priming is not well-understood, it is generally understood to involve specific physiological and biochemical reactions. ^26^^,^^27^ The synchronous germination and high germination rate seen as a result of seed priming is attributable to metabolic repair that occur during the priming process, ^28^ increase in the concentration of germination-promoting metabolites, ^26^ and osmotic fine-tuning. ^29^ Also, seed priming leads to an increase in the concentration of α-amylase, an enzyme that play huge role in starch metabolism, in order to meet the energy demand of the growing embryo. ^28^

Optimal priming duration is plant-specific due to seed structures that are unique morphologically and physiologically. Since bacteria secret substances that can promote the growth of plants, ^30^ the determination of effective concentration is also key towards the development of the wholesome application of these substances. Optimal concentrations of chemicals used in priming have been determined. ^31^^,^^32^

However, while there is ample amount of research works existing on other priming methods, microbial priming is less-well research and the optimization of priming parameters is even less so. Hence, we set out to understand the impact of steeping duration and metabolite concentration on the growth promotion activity of secondary metabolites on selected crops (cowpea, soybean, sesame, and okra) vis-a-vis some agro-morphic parameters by sowing seeds soaked in metabolites of some previously-isolated bacteria immediately without drying. The crops used for priming in this study are of huge economic importance in the tropical region where they are a vital source of dietary requirements. Therefore, there is the need to boost their production and seed priming is a veritable tool to achieving this end.

## Methods

### Test metabolites

The metabolites used for the study were extracted from cultures of *Serratia liquefaciens,* (OP830504), *S. liquefaciens* (OP830503), *Providencia rettgeri* (OP830491), *P. rettgeri* (OP830498), and *Bacillus cereus* (OP830501). The bacterial strains were isolated from rhizospheres within Afe Babalola University environment.

The bacterial strains were isolated using the standard pour-plating procedure. After isolation, distinct colonies were streaked on nutrient agar plates to obtain pure cultures, after which they were stored on agar slants at 4 °C±2 °C until when needed.

For metabolite extraction, the cold extraction method as reported by Ref. 33 was adopted. The respective extracts were put in clean sterile universal bottles and stored 4 °C±2 °C until when needed.

Characterization of the metabolites was carried using gas chromatography mass-spectroscopy procedure. The equipment (Varian 3800/4000) was equipped with an Agilent splitter split/splitless BP5 (30 m × 0.25 mm × 0.25 microns) capillary column with nitrogen used as a gas carrier.

### Test seeds

The seeds used for the study were cowpea ( *Vigna unguiculata*), soy-bean ( *Glycine max*), sorghum ( *Sorghum bicolor*), sesame ( *Sesamum indicum*), and okra ( *Abelmoschus esculentus*). All the seeds were sourced from local markets in Ado-Ekiti, Ekiti State, Nigeria.

Prior to use, the respective seeds were subjected to viability tests. Preliminary viability testing was carried out by soaking approximately 100 seeds from a lot in 200 mL of sterile distilled in a 400 mL beaker and allowed to stand for 2 min. Seeds that floated were discarded and considered non-viable while those that settled at the bottom of the beaker were subjected to further viability testing. Further viability testing of the seeds was carried out by planting approximately seven seeds in transparent plastic containers that contained absorbent cotton wool as blotters in triplicates and incubating under fluorescent light for 7 d. Seed lots that showed average percent germination of at least 65% were considered as viable and used for germinability studies.

### Germinability studies

Germinability experiments were carried out by investigating the effects of steeping duration and metabolite concentration on the seeds.

The effect of steeping duration of seeds in the respective metabolites on germinability was carried out under 1, 2, 3, 4, and 5 h. The respective seeds from the viable lots were steeped in a known concentration of metabolite and allowed to stand for 5 h duration. Every one hour, for a 5 h duration, approximately seven seeds were withdrawn and planted in transparent plastic cups and incubated for 7 d. At the expiration of incubation, final percent germination, mean germination time, germination index and vigor index were estimated as follows:

•
$\text{Final germination}\%\;\left(\mathrm{FGP}\right)=\frac{\text{total number of germinated seeds}}{\text{total number of seeds sown}}\;\times \;100\%$ ^34^
•
$\text{Mean germination time}\;\left(\mathrm{MGT}\right)=\sum \frac{\mathrm{fx}}{f}$ ^35^
  Where f is the number of seeds germinated on day x

•
$\text{Germination index}\;\left(\mathrm{GIX}\right)=\left(8\times N1\right)+\left(7\times N2\right)+\left(6\times N3\right)+\ldots +\left(1\times N8\right)$ ^36^
  Where N1 , N2,N3 … N8 represent the number of seeds that germinated on the first, second, and third until the 8th day, and 8, 9, 7 … 1 are the weights given to the number of germinated seeds on the first, second, and third day up to the 8th day.
•
$\text{Vigor index}\;\left(\mathrm{VIX}\right)=\mathrm{FGP}\times \text{average plant height}$ ^37^

With respect to effect of metabolite concentration, 200 mg/L, 400 mg/L, 600 mg/L, 800 mg/L and 1000 mg/L were used for the study. The seeds were steeped in the respective metabolite concentrations and allowed to stand for the optimal steeping time obtained in the first experiment before planting and incubation. At the expiration of the 7 day incubation period, final percent germination, mean germination time, germination index and vigor index were estimated were estimated.

### Ethical approval

The study did not involve humans or animals, hence ethical approval was not obtained from any ethics committee. The study proposal was however approved by the Board of the College of Postgraduate Studies, Afe Babalola University, Ado-Ekiti, Nigeria.

## Results

### Effect of steeping duration in metabolites

Generally, final percent germination of the cowpea seeds showed significantly highest and lowest values in setups steeped for 2 and 5 h, 1 and 5 h, 1, 2, and 3 and 4 h in metabolites from Isolates K, L and M, respectively. There was no significantly difference between final percent germination at the different steeping durations for seeds treated in metabolite from Isolate N. In addition, seeds treated with metabolite from Isolate O showed significantly lowest final percent germination at 3 and 5 h. With respect to mean germination time, significantly lowest values were recorded for seeds steeped for 1 h (metabolites from Isolates K and M), 4 and 5 h (metabolite from isolate L), 1, 4, and 5 h (metabolites from Isolate N), and 1, 2, and 4 (metabolite from Isolate O). Germination index showed significantly lowest values at 3 h (metabolite from Isolate L), 4 and 5 h (metabolite from Isolate M), 1-3 h (metabolite from Isolate N), and 3 and 5 h (metabolite from Isolate O). For vigor index, significantly lowest values were observed at 2, 4 and 5 h (metabolite from Isolate M), 1, 3, and 5 h (metabolite from Isolate N), and 4 and 5 h (metabolite from Isolate O). Also, germination and vigor indices of the seeds showed significantly lowest values in setups treated for 5 h (metabolites K and L) ( Table 1).

**Table 1.** Germinability of the cowpea seeds at the different steeping durations in the respective metabolites.

Parameter	Time	Isolate K	Isolate L	Isolate M	Isolate N	Isolate O
FPG	1 h	71.43 ^a^ (±0.00)	78.57 ^a^ (±7.82)	85.71 ^a^ (±0.00)	64.29 ^a^ (±7.82)	78.57 ^a^ (±7.82)
	2 h	85.71 ^b^ (±15.65)	64.29 ^b^ (±7.82)	71.43 ^ac^ (±15.65)	78.57 ^a^ (±7.82)	78.57 ^a^ (±7.82)
	3 h	71.43 ^a^ (±0.00)	42.86 ^c^ (±15.65)	71.43 ^ac^ (±31.30)	71.43 ^a^ (±0.00)	50.00 ^b^ (±23.47)
	4 h	71.43 ^a^ (±0.00)	57.14 ^b^ (±0.00)	50.00 ^b^ (±7.82)	78.57 ^a^ (±23.47)	78.57 ^a^ (±7.82)
	5 h	54.29 ^c^ (±15.65)	21.43 ^d^ (±7.82)	64.29 ^bc^ (±7.82)	78.57 ^a^ (±7.82)	64.29 ^ab^ (±23.47)
MGT	1 h	5.14 ^a^ (±0.05)	5.45 ^a^ (±0.25)	5.04 ^a^ (±0.04)	5.14 ^a^ (±0.06)	5.24 ^a^ (±0.06)
	2 h	5.51 ^b^ (±0.15)	5.37 ^ab^ (±0.01)	5.34 ^b^ (±0.02)	5.36 ^b^ (±0.08)	5.25 ^a^ (±0.17)
	3 h	5.32 ^c^ (±0.04)	5.23 ^b^ (±0.00)	5.39 ^b^ (±0.06)	5.40 ^b^ (±0.17)	5.40 ^b^ (±0.16)
	4 h	5.43 ^b^ (±0.08)	5.11 ^c^ (±0.00)	5.41 ^b^ (±0.10)	5.21 ^a^ (±0.11)	5.25 ^a^ (±0.07)
	5 h	5.38 ^bc^ (±0.08)	5.25 ^bc^ (±0.27)	5.57 ^c^ (±0.07)	5.24 ^a^ (±0.06)	5.45 ^b^ (±0.15)
GIX	1 h	129.50 ^a^ (±3.83)	120.00 ^a^ (±29.58)	164.50 ^a^ (±3.83)	116.00 ^ab^ (±18.62)	133.50 ^a^ (±8.22)
	2 h	125.00 ^a^ (±33.96)	101.50 ^a^ (±11.50)	115.50 ^b^ (±26.84)	124.00 ^ab^ (±18.62)	133.00 ^a^ (±0.00)
	3 h	116.00 ^a^ (±3.29)	73.50 ^b^ (±26.84)	112.50 ^b^ (±53.13)	110.00 ^b^ (±10.95)	79.00 ^b^ (±44.91)
	4 h	109.00 ^a^ (±4.38)	105.00 ^a^ (±0.00)	77.00 ^c^ (±7.67)	133.50 ^a^ (±31.22)	131.00 ^a^ (±9.86)
	5 h	84.40 ^b^ (±19.72)	35.00 ^c^ (±7.67)	89.50 ^bc^ (±16.98)	133.50 ^a^ (±8.22)	93.00 ^b^ (±25.20)
VIX	1 h	818.88 ^a^ (±69.86)	862.35 ^a^ (±163.31)	1578.37 ^a^ (±104.63)	783.78 ^a^ (±215.62)	943.06 ^ab^ (±2.46)
	2 h	1212.14 ^b^ (±240.22)	575.61 ^b^ (±130.67)	697.76 ^bc^ (±202.77)	1390.10 ^b^ (±258.55)	1064.49 ^a^ (±77.35)
	3 h	788.78 ^a^ (±32.42)	323.67 ^c^ (±239.21)	888.57 ^c^ (±625.97)	700.51 ^a^ (±100.04)	608.47 ^b^ (±535.09)
	4 h	877.55 ^a^ (±125.19)	507.35 ^b^ (±66.62)	343.98 ^b^ (±156.16)	1305.20 ^b^ (±800.23)	556.43 ^c^ (±266.37)
	5 h	434.12 ^c^ (±247.37)	37.55 ^d^ (±29.51)	353.88 ^b^ (±68.41)	999.18 ^ab^ (±229.37)	466.53 ^c^ (±347.41)

Values are averages of six replicates. Values in parenthesis represent ± standard deviations of means. Same and different superscripts on same column for a particular factor connote no significant difference and significant difference, respectively. K, L, M, N, and O represent *Serratia liquefaciens,* (OP830504), *S. liquefaciens* (OP830503), *Providencia rettgeri* (OP830491), *P. rettgeri* (OP830498), and *Bacillus cereus* (OP830501). FPG = final percent germination, MGT = mean germination time, GIX = germination index, and VIX = vigor index.

In the case of the soybean seeds, final percent germination of seeds showed the highest values at 2 h steeping duration when treated in the respective metabolites, apart from those treated in Isolate O, where 1 h steeping duration was observed to show highest values. Furthermore, significantly lowest mean germination times were recorded in seeds steeped for 1, 2, and 3 h, 1 and 2 h, 1, 4, and 5 h in metabolites from isolates L, M and N, respectively, and 2 h in metabolites from isolate K and 1, 3, and 4 h in metabolites from isolate O. However, significantly highest germination index was observed in seeds treated for 1 and 2 h in metabolite K, 2 h in metabolite L, 1-3 h in metabolite M, 2 and 3 h in metabolite N, and 1 h in metabolite O. Also, for seedling vigor index, significantly highest values were observed at 2 h (metabolites K and L), 1, 3, 4, and 5 h (metabolite M), 2 and 3 h (metabolite N), and 1 h (metabolite O) ( Table 2).

**Table 2.** Germinability of the soybean seeds at the different steeping durations in the respective metabolites.

Parameter	Time	Isolate K	Isolate L	Isolate M	Isolate N	Isolate O
FPG	1 h	64.29 ^a^ (±7.82)	50.00 ^ad^ (±7.82)	57.14 ^ac^ (±15.65)	50.00 ^a^ (±7.82)	85.71 ^a^ (±0.00)
	2 h	71.43 ^a^ (±15.65)	64.29 ^b^ (±7.82)	71.43 ^b^ (±0.00)	78.57 ^b^ (±7.82)	57.14 ^bc^ (±0.00)
	3 h	42.86 ^b^ (±15.65)	42.86 ^a^ (±0.00)	71.43 ^b^ (±0.00)	71.43 ^b^ (±15.65)	50.00 ^b^ (±7.82)
	4 h	57.14 ^ab^ (±15.65)	57.14 ^bd^ (±0.00)	50.00 ^a^ (±7.82)	57.14 ^a^ (±0.00)	50.00 ^b^ (±7.82)
	5 h	57.14 ^ab^ (±0.00)	50.00 ^ad^ (±7.82)	64.29 ^bc^ (±7.82)	50.00 ^a^ (±7.82)	64.29 ^c^ (±7.82)
MGT	1 h	5.37 ^a^ (±0.01)	5.34 ^a^ (±0.02)	5.31 ^a^ (±0.14)	5.50 ^a^ (±0.25)	5.51 ^ab^ (±0.21)
	2 h	5.27 ^b^ (±0.05)	5.44 ^ac^ (±0.06)	5.37 ^a^ (±0.10)	5.69 ^b^ (±0.10)	5.61 ^a^ (±0.12)
	3 h	5.67 ^c^ (±0.06)	5.41 ^ac^ (±0.10)	5.53 ^b^ (±0.15)	5.72 ^b^ (±0.15)	5.34 ^b^ (±0.12)
	4 h	5.48 ^d^ (±0.02)	5.65 ^b^ (±0.04)	5.55 ^b^ (±0.06)	5.61 ^ab^ (±0.12)	5.52 ^ab^ (±0.23)
	5 h	5.50 ^e^ (±0.00)	5.50 ^c^ (±0.20)	5.60 ^b^ (±0.01)	5.50 ^a^ (±0.00)	5.54 ^a^ (±0.08)
GIX	1 h	101.50 ^ab^ (±11.50)	80.50 ^a^ (±11.50)	95.00 ^ac^ (±33.96)	71.50 ^a^ (±0.55)	124.50 ^a^ (±16.98)
	2 h	119.00 ^b^ (±23.00)	98.00 ^b^ (±15.34)	112.50 ^a^ (±7.12)	99.00 ^b^ (±4.38)	78.00 ^bc^ (±6.57)
	3 h	57.00 ^c^ (±23.00)	66.50 ^c^ (±3.83)	101.00 ^ac^ (±12.05)	91.00 ^bc^ (±27.39)	81.00 ^bd^ (±18.62)
	4 h	84.50 ^a^ (±23.55)	75.50 ^ac^ (±2.74)	70.50 ^b^ (±8.22)	78.00 ^ac^ (±6.57)	71.00 ^b^ (±1.10)
	5 h	84.00 ^a^ (±0.00)	71.50 ^ac^ (±1.64)	88.50 ^c^ (±11.50)	73.50 ^a^ (±11.50)	92.00 ^cd^ (±15.34)
VIX	1 h	490.00 ^a^ (±60.14)	326.63 ^a^ (±28.50)	607.45 ^a^ (±339.48)	241.73 ^ad^ (±85.74)	1027.96 ^a^ (±329.30)
	2 h	926.73 ^b^ (±416.05)	593.67 ^b^ (±120.95)	845.41 ^b^ (±39.68)	897.14 ^b^ (±367.98)	494.69 ^b^ (±66.17)
	3 h	243.67 ^a^ (±223.11)	252.24 ^a^ (±91.88)	805.10 ^ab^ (±60.36)	711.63 ^bc^ (±437.06)	418.88 ^b^ (±196.62)
	4 h	474.59 ^a^ (±374.35)	305.71 ^a^ (±75.56)	422.45 ^a^ (±135.48)	556.73 ^c^ (±15.20)	243.67 ^c^ (±44.26)
	5 h	346.94 ^a^ (±76.01)	279.08 ^a^ (±85.06)	440.00 ^a^ (±110.89)	232.96 ^d^ (±115.02)	494.59 ^b^ (±132.24)

Values are averages of six replicates. Values in parenthesis represent ± standard deviations of means. Same and different superscripts on same column for a particular factor connote no significant difference and significant difference, respectively. K, L, M, N, and O represent *Serratia liquefaciens,* (OP830504), *S. liquefaciens* (OP830503), *Providencia rettgeri* (OP830491), *P. rettgeri* (OP830498), and *Bacillus cereus* (OP830501). FPG = final percent germination, MGT = mean germination time, GIX = germination index, and VIX = vigor index.

For the sesame seeds, remarkably high final percent germination values (>78%) were observed in the respective treatments, irrespective of the steeping duration. However, significantly lowest mean germination time was observed for seeds steeped for 1 h (metabolites from isolate O), 2 h (metabolites from isolates L and M) and 1, 2, 3, and 5 h (metabolites from isolate K). Also, significantly highest germination index was observed for seeds steeped for 2 and 4 h (metabolite from isolate K), 2 h (metabolites from isolates L and M) and 1 h (metabolites from isolates N and O). In the case of seedling vigor index, seeds steeped for 2 and 3 h (metabolites from isolates K and O), 1 h (metabolite from isolates L and M) and 3 h (metabolite from isolate N) showed significantly highest values ( Table 3).

**Table 3.** Germinability of the sesame seeds at the different steeping durations in the respective metabolites.

Parameter	Time	Isolate K	Isolate L	Isolate M	Isolate N	Isolate O
FPG	1 h	92.86 ^a^ (±7.82)	100.00 ^a^ (±0.00)	100.00 ^a^ (±0.00)	92.86 ^a^ (±7.82)	100.00 ^a^ (±0.00)
	2 h	100.00 ^a^ (±0.00)	100.00 ^a^ (±0.00)	100.00 ^a^ (±0.00)	100.00 ^b^ (±0.00)	100.00 ^a^ (±0.00)
	3 h	92.86 ^a^ (±7.82)	85.71 ^b^ (±0.00)	92.86 ^b^ (±7.82)	100.00 ^b^ (±0.00)	92.86 ^b^ (±7.82)
	4 h	100.00 ^a^ (±0.00)	78.57 ^c^ (±7.82)	100.00 ^a^ (±0.00)	100.00 ^b^ (±0.00)	92.86 ^b^ (±7.82)
	5 h	92.86 ^a^ (±7.82)	100.00 ^a^ (±0.00)	92.86 ^b^ (±7.82)	100.00 ^b^ (±0.00)	100.00 ^a^ (±0.00)
MGT	1 h	5.33 ^ab^ (±0.14)	5.42 ^a^ (±0.00)	5.30 ^a^ (±0.04)	5.00 ^a^ (±0.00)	5.16 ^a^ (±0.04)
	2 h	5.27 ^ab^ (±0.08)	5.20 ^b^ (±0.00)	5.13 ^b^ (±0.15)	5.42 ^a^ (±0.00)	5.38 ^b^ (±0.04)
	3 h	5.25 ^a^ (±0.02)	5.27 ^c^ (±0.05)	5.37 ^a^ (±0.06)	5.34 ^a^ (±0.00)	5.37 ^b^ (±0.04)
	4 h	5.34 ^b^ (±0.00)	5.35 ^d^ (±0.04)	5.34 ^a^ (±0.08)	5.34 ^a^ (±0.00)	5.53 ^c^ (±0.03)
	5 h	5.33 ^ab^ (±0.01)	5.34 ^d^ (±0.08)	5.50 ^c^ (±0.00)	5.42 ^a^ (±0.00)	5.38 ^b^ (±0.04)
GIX	1 h	148.50 ^a^ (±0.55)	154.00 ^a^ (±0.00)	164.50 ^a^ (±3.83)	182.00 ^a^ (±15.34)	178.50 ^a^ (±3.83)
	2 h	168.00 ^b^ (±7.67)	175.00 ^b^ (±0.00)	182.00 ^b^ (±15.34)	154.00 ^b^ (±0.00)	157.50 ^b^ (±3.83)
	3 h	157.50 ^a^ (±11.50)	143.50 ^c^ (±3.83)	147.00 ^c^ (±7.67)	161.00 ^b^ (±0.00)	147.00 ^c^ (±15.34)
	4 h	161.00 ^b^ (±0.00)	126.00 ^d^ (±15.34)	161.00 ^a^ (±7.67)	161.00 ^b^ (±0.00)	133.50 ^d^ (±8.22)
	5 h	150.50 ^a^ (±11.50)	161.00 ^a^ (±7.67)	136.50 ^c^ (±11.50)	154.00 ^b^ (±0.00)	157.50 ^b^ (±3.83)
VIX	1 h	528.67 ^a^ (±111.00)	800.00 ^a^ (±178.40)	952.86 ^a^ (±7.82)	666.53 ^a^ (±93.00)	756.43 ^a^ (±71.20)
	2 h	711.43 ^b^ (±12.52)	660.00 ^b^ (±115.80)	887.86 ^a^ (±2.35)	506.43 ^bd^ (±44.60)	877.86 ^b^ (±28.95)
	3 h	678.16 ^b^ (±33.31)	615.31 ^b^ (±2.01)	721.63 ^b^ (±42.48)	829.29 ^c^ (±97.81)	791.43 ^ab^ (±78.25)
	4 h	592.86 ^a^ (±31.30)	339.18 ^c^ (±22.80)	582.86 ^c^ (±50.08)	553.57 ^b^ (±44.60)	447.55 ^c^ (±138.83)
	5 h	511.73 ^a^ (±95.12)	455.71 ^d^ (±0.00)	461.73 ^d^ (±106.08)	450.71 ^d^ (±22.69)	496.43 ^c^ (±44.60)

Values are averages of six replicates. Values in parenthesis represent ± standard deviations of means. Same and different superscripts on same column for a particular factor connote no significant difference and significant difference, respectively. K, L, M, N, and O represent *Serratia liquefaciens,* (OP830504), *S. liquefaciens* (OP830503), *Providencia rettgeri* (OP830491), *P. rettgeri* (OP830498), and *Bacillus cereus* (OP830501). FPG = final percent germination, MGT = mean germination time, GIX = germination index, and VIX = vigor index.

When okra seeds were steeped in the metabolites for varying durations, final percent germination of the okra seeds showed significantly highest values in seeds treated for 3 h (metabolite K), 4 h (metabolite L) and 1 and 5 h (metabolite N). There was no significant difference between final germination of the seeds treated with metabolite from isolate M at the different steeping durations. Also, mean germination time showed no significant difference at the different steeping durations for seeds treated with metabolites from isolates K, M and O. However, significantly lowest mean germination times were observed for seeds steeped for 2 and 5 h and 3-5 h, when treated with metabolites from isolates L and N, respectively. Furthermore, seeds steeped for 3 h showed significantly highest germination and vigor indices when treated with metabolites from isolates K respectively. Significantly highest germination index values were observed for seeds steeped for 3 h (Metabolite K), 2-4 h (metabolite L), 1, 3, and 4 h (metabolite M), 1, 3, 4, and 5 h (Isolate N), and 1-4 h (Isolate O). Finally, for seedling vigor index, significantly highest values were observed at 3 h (metabolite K), 3 and 4 h (metabolite L), 3 and 4 h (metabolite M), 1, 4, and 5 (metabolite N), and 3 and 4 h (metabolite O) ( Table 4).

**Table 4.** Germinability of the okra seeds at the different steeping durations in the respective metabolites.

Parameter	Time	Isolate K	Isolate L	Isolate M	Isolate N	Isolate O
FPG	1 h	28.57 ^a^ (±15.65)	57.14 ^ad^ (±15.65)	85.71 ^a^ (±0.00)	85.71 ^a^ (±0.00)	71.43 ^ab^ (±15.65)
	2 h	71.43 ^b^ (±0.00)	71.43 ^ab^ (±0.00)	78.57 ^a^ (±7.82)	64.29 ^b^ (±7.82)	71.43 ^ab^ (±15.65)
	3 h	85.71 ^c^ (±0.00)	85.71 ^bc^ (±0.00)	78.57 ^a^ (±7.82)	71.43 ^bc^ (±0.00)	85.71 ^a^ (±15.65)
	4 h	64.29 ^b^ (±7.82)	92.86 ^c^ (±7.82)	78.57 ^a^ (±7.82)	64.29 ^b^ (±23.47)	78.57 ^ab^ (±7.82)
	5 h	71.43 ^b^ (±15.65)	50.00 ^d^ (±23.47)	78.57 ^a^ (±23.47)	78.57 ^ac^ (±7.82)	64.29 ^b^ (±7.82)
MGT	1 h	5.41 ^a^ (±0.10)	5.28 ^ac^ (±0.01)	5.30 ^a^ (±0.08)	5.47 ^a^ (±0.20)	5.22 ^a^ (±0.24)
	2 h	5.33 ^a^ (±0.16)	5.14 ^bd^ (±0.05)	5.39 ^a^ (±0.33)	5.91 ^b^ (±0.06)	5.29 ^a^ (±0.23)
	3 h	5.29 ^a^ (±0.07)	5.36 ^a^ (±0.02)	5.30 ^a^ (±0.13)	5.35 ^c^ (±0.04)	5.28 ^a^ (±0.30)
	4 h	5.33 ^a^ (±0.11)	5.39 ^a^ (±0.06)	5.19 ^a^ (±0.01)	5.27 ^c^ (±0.05)	5.20 ^a^ (±0.05)
	5 h	5.28 ^a^ (±0.31)	5.19 ^cd^ (±0.21)	5.38 ^a^ (±0.17)	5.34 ^c^ (±0.02)	5.35 ^a^ (±0.26)
GIX	1 h	45.50 ^a^ (±26.84)	93.00 ^a^ (±24.10)	140.50 ^a^ (±7.12)	124.00 ^a^ (±18.62)	120.00 ^ab^ (±8.76)
	2 h	114.00 ^b^ (±13.15)	129.50 ^b^ (±3.83)	121.00 ^bc^ (±13.15)	71.00 ^b^ (±5.48)	122.50 ^ab^ (±42.17)
	3 h	141.00 ^c^ (±6.57)	133.00 ^b^ (±1.10)	127.50 ^abd^ (±1.64)	113.00 ^a^ (±1.10)	139.00 ^ab^ (±1.10)
	4 h	102.50 ^b^ (±4.93)	144.50 ^b^ (±18.07)	137.00 ^ab^ (±12.05)	108.50 ^a^ (±42.17)	137.00 ^a^ (±18.62)
	5 h	114.50 ^b^ (±2.74)	84.00 ^a^ (±30.67)	120.00 ^cd^ (±24.10)	126.50 ^a^ (±14.79)	102.00 ^b^ (±3.29)
VIX	1 h	108.27 ^a^ (±92.67)	335.31 ^a^ (±154.70)	580.41 ^ac^ (±45.61)	743.27 ^a^ (±73.77)	488.98 ^ac^ (±140.40)
	2 h	495.92 ^bd^ (±36.89)	557.65 ^b^ (±73.22)	539.29 ^ad^ (±126.87)	303.88 ^b^ (±9.17)	522.65 ^ac^ (±230.94)
	3 h	869.39 ^c^ (±8.05)	836.33 ^c^ (±12.07)	643.88 ^ab^ (±133.02)	511.22 ^c^ (±4.47)	818.88 ^b^ (±292.31)
	4 h	537.45 ^b^ (±112.12)	754.08 ^c^ (±109.77)	713.67 ^b^ (±61.93)	553.47 ^ac^ (±336.68)	710.20 ^ab^ (±140.84)
	5 h	410.82 ^d^ (±171.02)	250.41 ^a^ (±222.00)	488.47 ^cd^ (±104.96)	559.90 ^ac^ (±100.27)	392.55 ^c^ (±100.94)

Values are averages of six replicates. Values in parenthesis represent ± standard deviations of means. Same and different superscripts on same column for a particular factor connote no significant difference and significant difference, respectively. K, L, M, N, and O represent *Serratia liquefaciens,* (OP830504), *S. liquefaciens* (OP830503), *Providencia rettgeri* (OP830491), *P. rettgeri* (OP830498), and *Bacillus cereus* (OP830501). FPG = final percent germination, MGT = mean germination time, GIX = germination index, and VIX = vigor index.

### Effect of concentration of metabolite

At the different concentrations of the respective metabolites, significantly lowest final precent germination values were observed for cowpea seeds treated with 800 mg/L of metabolites from isolates K, L, and N. Final percent germination of cowpea seeds treated with metabolites from isolates M and O did not differ significantly between the respective concentrations. Also, significantly lowest mean germination time was observed for seeds treated with metabolite concentrations of 200, 600, and 800 mg/L (metabolite from isolate K), 400 and 800 mg/L (metabolite from isolate L), and 400 mg/L (metabolite from isolate s M), 200 – 800 mg/L (metabolite N), and 200-600 mg/L (metabolite O). Significantly highest germination index values were observed in seeds treated with metabolite concentrations of 200 and 600 mg/L (metabolite K), 600 mg/L (metabolite L), 200, 400, 600, and 1000 mg/L (metabolite N), and 200, 600, and 800 mg/L (metabolite O) ( Table 5). In the case of seedling vigor index, significantly highest values were observed at concentrations of 600 mg/L (metabolite K), 200, 600, and 1000 mg/L (metabolite L), 600 and 1000 mg/L (metabolite M), 400, 600, and 1000 mg/L (metabolite N), and 200-600 mg/L (metabolite O) ( Table 5).

**Table 5.** Germinability of the cowpea seeds at the different concentrations of the respective metabolites.

Parameter	Concentration	Isolate K	Isolate L	Isolate M	Isolate N	Isolate O
FPG	200 mg/L	71.43 ^ab^ (±0.00)	64.29 ^a^ (±23.47)	50.00 ^a^ (±7.82)	64.29 ^a^ (±23.47)	64.29 ^a^ (±7.82)
	400 mg/L	57.14 ^ac^ (±31.30)	42.86 ^b^ (±0.00)	42.86 ^a^ (±0.00)	85.71 ^b^ (±0.00)	57.14 ^a^ (±46.95)
	600 mg/L	85.71 ^b^ (±0.00)	85.71 ^c^ (±0.00)	57.14 ^a^ (±31.30)	85.71 ^b^ (±15.65)	78.57 ^a^ (±7.82)
	800 mg/L	50.00 ^c^ (±7.82)	14.29 ^d^ (±0.00)	50.00 ^a^ (±7.82)	42.86 ^a^ (±0.00)	64.29 ^a^ (±7.82)
	1000 mg/L	71.43 ^abc^ (±15.65)	64.29 ^a^ (±23.47)	57.14 ^a^ (±0.00)	78.57 ^ab^ (±23.47)	71.43 ^a^ (±15.65)
MGT	200 mg/L	5.32 ^a^ (±0.25)	5.67 ^a^ (±0.09)	5.61 ^a^ (±0.12)	5.45 ^a^ (±0.05)	5.37 ^ab^ (±0.01)
	400 mg/L	5.62 ^b^ (±0.12)	5.38 ^bc^ (±0.07)	5.25 ^b^ (±0.27)	5.47 ^ab^ (±0.11)	5.28 ^a^ (±0.31)
	600 mg/L	5.39 ^ab^ (±0.09)	5.47 ^b^ (±0.08)	5.54 ^a^ (±0.04)	5.47 ^ab^ (±0.05)	5.40 ^ac^ (±0.24)
	800 mg/L	5.52 ^ab^ (±0.23)	5.25 ^c^ (±0.27)	5.69 ^a^ (±0.04)	5.41 ^a^ (±0.10)	5.52 ^bc^ (±0.07)
	1000 mg/L	5.64 ^b^ (±0.35)	5.51 ^ab^ (±0.01)	5.55 ^a^ (±0.06)	5.56 ^b^ (±0.07)	5.75 ^d^ (±0.16)
GIX	200 mg/L	116.50 ^a^ (±18.07)	84.00 ^a^ (±35.05)	67.50 ^a^ (±4.93)	98.00 ^a^ (±38.34)	101.50 ^ab^ (±11.50)
	400 mg/L	79.00 ^b^ (±47.10)	67.00 ^a^ (±3.29)	73.50 ^a^ (±11.50)	127.00 ^a^ (±7.67)	84.50 ^a^ (±61.89)
	600 mg/L	133.50 ^a^ (±7.12)	127.00 ^b^ (±6.57)	81.00 ^a^ (±42.72)	125.50 ^a^ (±26.84)	120.50 ^b^ (±6.02)
	800 mg/L	71.00 ^b^ (±1.10)	24.50 ^c^ (±3.83)	64.50 ^a^ (±8.22)	66.50 ^b^ (±3.83)	92.00 ^ab^ (±7.67)
	1000 mg/L	88.50 ^b^ (±3.83)	92.50 ^a^ (±32.32)	81.00 ^a^ (±3.29)	109.50 ^a^ (±27.93)	85.50 ^a^ (±8.22)
VIX	200 mg/L	675.00 ^a^ (±123.52)	584.69 ^ab^ (±234.07)	318.98 ^a^ (±122.73)	741.12 ^a^ (±481.21)	765.92 ^a^ (±137.94)
	400 mg/L	588.98 ^ac^ (±540.57)	308.57 ^ac^ (±42.92)	331.53 ^a^ (±147.89)	1279.59 ^b^ (±34.88)	1042.55 ^a^ (±1097.35)
	600 mg/L	1100.20 ^b^ (±10.06)	918.37 ^b^ (±490.94)	719.18 ^b^ (±627.31)	1406.33 ^b^ (±612.78)	1065.82 ^a^ (±254.30)
	800 mg/L	320.92 ^c^ (±27.61)	16.94 ^c^ (±0.22)	330.41 ^a^ (±73.55)	302.76 ^a^ (±66.73)	796.73 ^b^ (±137.71)
	1000 mg/L	525.31 ^ac^ (±178.40)	744.49 ^ab^ (±618.37)	411.84 ^ab^ (±97.03)	1240.41 ^b^ (±443.99)	863.06 ^b^ (±236.30)

Values are averages of six replicates. Values in parenthesis represent ± standard deviations of means. Same and different superscripts on same column for a particular factor connote no significant difference and significant difference, respectively. K, L, M, N, and O represent *Serratia liquefaciens,* (OP830504), *S. liquefaciens* (OP830503), *Providencia rettgeri* (OP830491), *P. rettgeri* (OP830498), and *Bacillus cereus* (OP830501). FPG = final percent germination, MGT = mean germination time, GIX = germination index, and VIX = vigor index.

For the soybean seeds, significantly lowest final percent germination values were observed in treatments with metabolite concentrations of 200, 600, 800, and 1000 mg/L (metabolite from isolate K), 800 mg/L (metabolite from isolate M), and 600 mg/L (metabolite from isolate from N), and 1000 mg/L (metabolite from isolate O). In the case of mean germination time, significantly lowest values were recorded in seeds that were treated with 200, 400, and 800 mg/L (metabolite from isolate K), 200, 400, 600, and 1000 mg/L (metabolite from isolate L), 200, 600, and 1000 mg/L (metabolite from isolate M), 200, 400, and 800 mg/L (metabolite from isolate N), 200, 400, and 600 mg/L (metabolite from isolate M). Generally, highest germination index values were observed in seeds that were treated with 400 mg/L, 600 and 1000 mg/L, 400 and 800 mg/L, and 400 and 800 mg/L of metabolites from isolates K, M, N, and O, respectively.

For seeds treated with metabolites from isolates K, M, N, and O, significantly highest seedling vigor index values were observed in seeds treated with 400 mg/L, 600 mg/L, 400 mg/L, and 600 mg/L, respectively ( Table 6).

**Table 6.** Germinability of the soybean seeds at the different concentrations of the respective metabolites.

Parameter	Concentration	Isolate K	Isolate L	Isolate M	Isolate N	Isolate O
FPG	200 mg/L	42.86 ^ab^ (±15.65)	42.86 ^a^ (±31.30)	71.43 ^a^ (±15.65)	50.00 ^ac^ (±7.82)	50.00 ^a^ (±7.82)
	400 mg/L	50.00 ^a^ (±7.82)	42.86 ^a^ (±31.30)	64.29 ^a^ (±7.82)	50.00 ^ad^ (±7.82)	57.14 ^a^ (±15.65)
	600 mg/L	42.86 ^ab^ (±0.00)	42.86 ^a^ (±15.65)	85.71 ^b^ (±0.00)	14.29 ^b^ (±0.00)	85.71 ^b^ (±0.00)
	800 mg/L	35.71 ^b^ (±7.82)	35.71 ^a^ (±23.47)	42.86 ^c^ (±15.65)	57.14 ^a^ (±0.00)	71.43 ^c^ (±0.00)
	1000 mg/L	42.86 ^ab^ (±15.65)	42.86 ^a^ (±15.65)	85.71 ^d^ (±0.00)	42.86 ^cd^ (±15.65)	35.71 ^d^ (±7.82)
MGT	200 mg/L	5.43 ^a^ (±0.08)	5.50 ^a^ (±0.00)	5.52 ^ab^ (±0.00)	5.59 ^ab^ (±0.10)	5.39 ^a^ (±0.08)
	400 mg/L	5.41 ^a^ (±0.10)	5.59 ^ab^ (±0.10)	5.55 ^a^ (±0.10)	5.55 ^a^ (±0.06)	5.35 ^a^ (±0.04)
	600 mg/L	5.66 ^bc^ (±0.17)	5.59 ^ab^ (±0.10)	5.47 ^b^ (±0.00)	5.75 ^bcd^ (±0.27)	5.36 ^a^ (±0.05)
	800 mg/L	5.52 ^ab^ (±0.31)	5.93 ^b^ (±0.62)	5.55 ^a^ (±0.06)	5.61 ^ad^ (±0.00)	5.54 ^b^ (±0.05)
	1000 mg/L	5.73 ^c^ (±0.00)	5.68 ^ab^ (±0.24)	5.51 ^ab^ (±0.01)	5.81 ^c^ (±0.09)	5.59 ^b^ (±0.15)
GIX	200 mg/L	66.50 ^ab^ (±26.84)	63.00 ^a^ (±46.01)	101.00 ^a^ (±23.00)	68.00 ^a^ (±5.48)	77.50 ^a^ (±8.22)
	400 mg/L	77.00 ^b^ (±7.67)	57.00 ^a^ (±39.44)	89.50 ^a^ (±4.93)	70.50 ^ac^ (±8.22)	91.00 ^ac^ (±23.00)
	600 mg/L	57.00 ^a^ (±6.57)	57.50 ^a^ (±16.98)	127.00 ^b^ (±0.00)	18.00 ^b^ (±3.29)	136.50 ^b^ (±3.83)
	800 mg/L	48.50 ^a^ (±0.55)	50.50 ^a^ (±44.37)	60.00 ^c^ (±19.72)	78.00 ^c^ (±0.00)	102.00 ^c^ (±3.29)
	1000 mg/L	54.00 ^a^ (±19.72)	58.00 ^a^ (±29.58)	124.00 ^b^ (±2.19)	49.00 ^d^ (±14.24)	50.00 ^d^ (±15.34)
VIX	200 mg/L	237.35 ^a^ (±216.63)	153.98 ^a^ (±133.13)	847.55 ^ac^ (±398.16)	305.10 ^a^ (±53.88)	439.29 ^a^ (±98.25)
	400 mg/L	390.10 ^b^ (±115.47)	355.31 ^a^ (±351.88)	581.94 ^ad^ (±139.39)	183.27 ^b^ (±62.15)	450.61 ^a^ (±234.07)
	600 mg/L	146.94 ^a^ (±12.74)	238.17 ^a^ (±160.29)	2450.20 ^b^ (±1569.39)	25.31 ^c^ (±17.44)	1251.43 ^b^ (±29.51)
	800 mg/L	154.29 ^a^ (±32.19)	304.90 ^a^ (±317.90)	266.12 ^a^ (±202.99)	319.59 ^a^ (±68.41)	703.57 ^c^ (±124.63)
	1000 mg/L	149.18 ^a^ (±68.19)	280.82 ^a^ (±229.82)	1249.59 ^cd^ (±98.59)	62.45 ^c^ (±11.18)	180.82 ^d^ (±50.08)

Values are averages of six replicates. Values in parenthesis represent ± standard deviations of means. Same and different superscripts on same column for a particular factor connote no significant difference and significant difference, respectively. K, L, M, N, and O represent *Serratia liquefaciens,* (OP830504), *S. liquefaciens* (OP830503), *Providencia rettgeri* (OP830491), *P. rettgeri* (OP830498), and *Bacillus cereus* (OP830501). FPG = final percent germination, MGT = mean germination time, GIX = germination index, and VIX = vigor index.

In the case of the sesame seeds, remarkably high final germination (> 90 %) was observed at all steeping duration for all steeping durations. Significantly lowest mean germination time was recorded for seeds treated with 600 mg/L (metabolites from isolates K and L), 1000 mg/L (metabolites from isolates M and O) and 200, 400, and 600 mg/L (metabolite from isolate N). Generally, significantly highest germination index was observed in seeds treated with 600 mg/L of metabolite from isolate K, 600 mg/L, 800 mg/L, and 1000 mg/L of metabolites from isolate L, 1000 mg/L of metabolites from isolate M, 200-600 mg/L of metabolites from isolate N, and 1000 mg/L of metabolites from isolate O. For seedling vigor index, significantly highest values were observed at 400 and 600 mg/L for metabolite K, 600 and 800 mg/L for metabolite L, 200 and 1000 mg/L for metabolite M, 200 mg/L for metabolite N, and 1000 mg/L for metabolite O ( Table 7).

**Table 7.** Germinability of the sesame seeds at the different concentrations of the respective metabolites.

Parameter	Concentration	Isolate K	Isolate L	Isolate M	Isolate N	Isolate O
FPG	200 mg/L	96.43 ^ab^ (±3.91)	85.71 ^a^ (±0.00)	100.00 ^a^ (±0.00)	100.00 ^a^ (±0.00)	92.86 ^a^ (±7.82)
	400 mg/L	100.00 ^a^ (±0.00)	85.71 ^a^ (±0.00)	100.00 ^a^ (±0.00)	100.00 ^a^ (±0.00)	92.86 ^a^ (±7.82)
	600 mg/L	100.00 ^a^ (±0.00)	92.86 ^b^ (±7.82)	92.86 ^b^ (±7.82)	100.00 ^a^ (±0.00)	92.86 ^a^ (±7.82)
	800 mg/L	92.86 ^b^ (±7.82)	100.00 ^c^ (±0.00)	92.86 ^b^ (±7.82)	92.86 ^b^ (±7.82)	92.86 ^a^ (±7.82)
	1000 mg/L	92.86 ^b^ (±7.82)	100.00 ^c^ (±0.00)	100.00 ^a^ (±0.00)	100.00 ^a^ (±0.00)	100.00 ^a^ (±0.00)
MGT	200 mg/L	5.20 ^a^ (±0.00)	5.32 ^a^ (±0.13)	5.30 ^a^ (±0.04)	5.23 ^ac^ (±0.04)	5.21 ^ab^ (±0.02)
	400 mg/L	5.20 ^a^ (±0.08)	5.18 ^b^ (±0.03)	5.38 ^b^ (±0.04)	5.22 ^a^ (±0.05)	5.17 ^a^ (±0.11)
	600 mg/L	5.06 ^b^ (±0.07)	5.03 ^c^ (±0.03)	5.33 ^ab^ (±0.01)	5.22 ^a^ (±0.05)	5.21 ^ab^ (±0.06)
	800 mg/L	5.14 ^c^ (±0.01)	5.21 ^b^ (±0.09)	5.37 ^b^ (±0.06)	5.32 ^b^ (±0.10)	5.25 ^b^ (±0.02)
	1000 mg/L	5.17 ^ac^ (±0.03)	5.13 ^b^ (±0.00)	5.16 ^c^ (±0.04)	5.30 ^bc^ (±0.04)	5.13 ^c^ (±0.00)
GIX	200 mg/L	168.25 ^a^ (±7.39)	138.00 ^a^ (±10.95)	164.50 ^a^ (±3.83)	171.50 ^a^ (±3.83)	161.00 ^a^ (±15.34)
	400 mg/L	175.00 ^a^ (±7.67)	151.00 ^b^ (±3.29)	157.50 ^a^ (±3.83)	172.00 ^a^ (±4.38)	164.50 ^a^ (±3.83)
	600 mg/L	189.00 ^b^ (±7.67)	178.50 ^c^ (±11.50)	150.50 ^b^ (±11.50)	172.00 ^a^ (±4.38)	161.00 ^a^ (±7.67)
	800 mg/L	168.00 ^a^ (±15.34)	172.50 ^c^ (±10.41)	147.00 ^b^ (±7.67)	150.50 ^b^ (±3.83)	157.50 ^a^ (±11.50)
	1000 mg/L	164.50 ^a^ (±11.50)	182.00 ^c^ (±0.00)	178.50 ^d^ (±3.83)	164.50 ^c^ (±3.83)	182.00 ^b^ (±0.00)
VIX	200 mg/L	470.20 ^a^ (±54.55)	377.14 ^a^ (±59.02)	542.86 ^ad^ (±10.95)	529.29 ^a^ (±10.17)	437.96 ^a^ (±39.79)
	400 mg/L	603.57 ^b^ (±151.01)	380.20 ^a^ (±3.35)	458.57 ^bc^ (±104.85)	478.57 ^b^ (±28.17)	433.16 ^a^ (±37.22)
	600 mg/L	682.14 ^b^ (±66.51)	491.63 ^bc^ (±137.49)	450.82 ^c^ (±53.88)	445.71 ^b^ (±20.34)	455.51 ^a^ (±45.61)
	800 mg/L	470.00 ^a^ (±17.21)	409.29 ^ab^ (±44.60)	407.96 ^c^ (±25.71)	457.45 ^b^ (±35.66)	432.76 ^a^ (±22.02)
	1000 mg/L	437.86 ^a^ (±85.29)	567.86 ^c^ (±88.42)	525.00 ^d^ (±30.52)	450.71 ^b^ (±69.64)	524.29 ^b^ (±0.00)

Values are averages of six replicates. Values in parenthesis represent ± standard deviations of means. Same and different superscripts on same column for a particular factor connote no significant difference and significant difference, respectively. K, L, M, N, and O represent *Serratia liquefaciens,* (OP830504), *S. liquefaciens* (OP830503), *Providencia rettgeri* (OP830491), *P. rettgeri* (OP830498), and *Bacillus cereus* (OP830501). FPG = final percent germination, MGT = mean germination time, GIX = germination index, and VIX = vigor index.

Furthermore, final percent germination of the okra seeds showed significantly lowest values in setups that were treated with 800 mg/L of metabolites from isolates K and N, 200, 800, and 1000 mg/L of metabolite from isolate L, 800 and 1000 mg/L of metabolite from isolate M, and 200 mg/L of metabolite from isolate O. In the case of mean germination time, seeds treated with metabolites from isolates M and N showed no significance difference at the different concentrations, while 800 mg/L and 200, 400, and 1000 mg/L of metabolites from isolates K and O showed significantly highest values, respectively. With respect to germination index, significantly highest values were recorded in seeds treated with 400, 600, and 1000 mg/L (metabolite from isolate K), 600 mg/L (metabolite from isolate L), 200-800 mg/L (metabolite from isolate M), and 800 and 1000 mg/L (metabolites from isolates N and O). For vigor index, seeds treated in metabolites at 400, 600, and 1000 mg/L (metabolite K), 600 mg/L (metabolite L), 200-600 mg/L (metabolites M and N), and 800-1000 mg/L (metabolite K) ( Table 8).

**Table 8.** Germinability of the okra seeds at the different concentrations of the respective metabolites.

Parameter	Concentration	Isolate K	Isolate L	Isolate M	Isolate N	Isolate O
FPG	200 mg/L	28.57 ^a^ (±0.00)	28.57 ^a^ (±0.00)	42.86 ^a^ (±31.30)	42.86 ^a^ (±15.65)	28.57 ^a^ (±0.00)
	400 mg/L	42.86 ^b^ (±15.65)	50.00 ^b^ (±7.82)	42.86 ^a^ (±0.00)	42.86 ^a^ (±15.65)	42.86 ^b^ (±0.00)
	600 mg/L	42.86 ^b^ (±15.65)	64.29 ^c^ (±7.82)	42.86 ^a^ (±15.65)	28.57 ^b^ (±0.00)	35.71 ^c^ (±7.82)
	800 mg/L	7.14 ^c^ (±7.82)	28.57 ^a^ (±0.00)	28.57 ^ab^ (±0.00)	7.14 ^c^ (±7.82)	64.29 ^d^ (±7.82)
	1000 mg/L	50.00 ^b^ (±7.82)	35.71 ^a^ (±7.82)	14.29 ^b^ (±0.00)	64.29 ^d^ (±7.82)	71.43 ^e^ (±0.00)
MGT	200 mg/L	5.37 ^a^ (±0.15)	5.37 ^a^ (±0.15)	5.44 ^a^ (±0.06)	5.26 ^a^ (±0.17)	5.50 ^a^ (±0.00)
	400 mg/L	5.37 ^a^ (±0.15)	5.43 ^ab^ (±0.08)	5.41 ^a^ (±0.10)	5.67 ^a^ (±0.06)	5.50 ^a^ (±0.00)
	600 mg/L	5.23 ^a^ (±0.00)	5.54 ^b^ (±0.05)	5.50 ^a^ (±0.00)	5.86 ^a^ (±0.15)	5.41 ^b^ (±0.10)
	800 mg/L	5.75 ^b^ (±3.01)	5.37 ^a^ (±0.15)	5.50 ^a^ (±0.00)	5.75a (±3.01)	5.32 ^c^ (±0.04)
	1000 mg/L	5.27 ^a^ (±0.05)	5.44 ^ab^ (±0.23)	5.50 ^a^ (±0.00)	5.50 ^a^ (±0.00)	5.47 ^ab^ (±0.09)
GIX	200 mg/L	45.50 ^a^ (±3.83)	45.50 ^a^ (±3.83)	66.50 ^a^ (±49.84)	74.00 ^a^ (±33.96)	42.00 ^a^ (±0.00)
	400 mg/L	66.50 ^b^ (±19.17)	77.00 ^b^ (±15.34)	66.50 ^a^ (±3.83)	57.00 ^a^ (±23.00)	63.00 ^b^ (±0.00)
	600 mg/L	73.50 ^b^ (±26.84)	91.50 ^c^ (±8.22)	63.00 ^a^ (±23.00)	33.00 ^b^ (±3.29)	56.00 ^b^ (±15.34)
	800 mg/L	10.50 ^c^ (±11.50)	45.50 ^a^ (±3.83)	42.00 ^ab^ (±0.00)	10.50 ^bc^ (±11.50)	105.00 ^c^ (±15.34)
	1000 mg/L	84.00 ^b^ (±15.34)	53.00 ^a^ (±4.38)	21.00 ^b^ (±0.00)	94.50 ^c^ (±11.50)	106.00 ^c^ (±6.57)
VIX	200 mg/L	83.06 ^a^ (±9.61)	106.94 ^a^ (±20.12)	285.10 ^a^ (±275.65)	217.96 ^a^ (±124.30)	84.08 ^a^ (±16.54)
	400 mg/L	181.02 ^b^ (±105.74)	201.94 ^b^ (±2.12)	253.16 ^ab^ (±34.54)	174.08 ^ab^ (±76.68)	196.53 ^a^ (±55.00)
	600 mg/L	212.24 ^b^ (±126.09)	410.41 ^c^ (±160.74)	205.51 ^ab^ (±109.32)	93.06 ^ab^ (±15.20)	205.71 ^a^ (±81.82)
	800 mg/L	10.82 ^a^ (±11.85)	66.73 ^a^ (±14.53)	99.18 ^bc^ (±21.01)	15.20 ^b^ (±16.66)	479.39 ^b^ (±223.78)
	1000 mg/L	232.04 ^b^ (±70.42)	126.43 ^ab^ (±45.94)	29.69 ^c^ (±7.49)	621.53 ^c^ (±274.87)	589.80 ^b^ (±40.24)

Values are averages of six replicates. Values in parenthesis represent ± standard deviations of means. Same and different superscripts on same column for a particular factor connote no significant difference and significant difference, respectively. K, L, M, N, and O represent *Serratia liquefaciens,* (OP830504), *S. liquefaciens* (OP830503), *Providencia rettgeri* (OP830491), *P. rettgeri* (OP830498), and *Bacillus cereus* (OP830501). FPG = final percent germination, MGT = mean germination time, GIX = germination index, and VIX = vigor index.

### Detected compounds in the metabolites

In the metabolite from *S. liquefaciens* (OP830504), the major compounds that were detected included Methyl lactate (10.40%), 9,12-Octadecadienoic acid (Z,Z)- (17.50%), n-Hexadecanoic acid (13.38%), Phytol (5.96%), Oleic acid (11.48%) and 9,12-Octadecadienoic acid (Z,Z)- (17.01%). Also, for the metabolite from *P. rettgeri* (OP830491), n-Hexadecanoic acid (14.13%), Octadecane (7.90%), Phytol (9.31%), 11,14,17-Eicosatrienoic acid, methyl ester (5.96%), Lupeol (7.74%), Stigmasterol (15.00%) and β-Sitosterol (12.19%) were the most dominant compounds ( Table 9).

**Table 9.** Detected compounds in the metabolites.

Peak #	RT	Compound detected	Comp. wt%	RT	Compound detected	Comp. wt%
	**Metabolite from *S. liquefaciens* (OP830504)**			**Metabolite from *P. rettgeri* (OP830491)**		
1	2.50	Methyl lactate	10.40	2.54	Methyl lactate	2.28
2	2.99	Cyclohexanol, 5-methyl-2-(1-methylethyl)-	3.93	8.00	Cyclohexanol, 5-methyl-2-(1-methylethyl)-	5.00
3	4.46	Pentanoic acid, 2-methylbutyl ester	1.38	12.48	Pentanoic acid, 2-methylbutyl ester	2.51
4	8.50	9,12-Octadecadienoic acid (Z,Z)-	17.50	14.23	9,12-Octadecadienoic acid (Z,Z)-	2.07
5	9.52	Tetradecanoic acid	1.57	16.60	Tetradecanoic acid	2.55
6	15.15	Dibutyl phthalate	1.39	17.58	Dibutyl phthalate	1.97
7	16.00	9-Octadecenoic acid (Z)-, methyl ester	3.97	25.00	n-Hexadecanoic acid	14.13
8	21.00	3,7,11,15-tetramethyl-2-hexadecen-1-ol	1.29	30.00	3,7,11,15-tetramethyl-2-hexadecen-1-ol	1.52
9	25.00	Octadecane	2.03	31.97	Octadecane	7.90
10	32.00	n-Hexadecanoic acid	13.38	35.51	9-Octadecenoic acid (Z)-, methyl ester	1.69
11	34.50	Phytol	5.96	36.25	Phytol	9.31
12	35.81	Oleic acid	11.48	37.62	Oleic acid	2.98
13	37.52	9,12-Octadecadienoic acid (Z,Z)-	17.01	39.94	9,12-Octadecadienoic acid (Z,Z)-	1.25
14	39.00	11,14,17-Eicosatrienoic acid, methyl ester	3.89	41.00	11,14,17-Eicosatrienoic acid, methyl ester	5.96
15	41.98	Squalene	2.51	41.50	Lupeol	7.74
16	44.50	Stigmasterol	1.83	43.50	Stigmasterol	15.00
17				44.23	β-Sitosterol	12.19
18				44.99	Squalene	2.78
	**Metabolite from *S. liquefaciens* (OP830503)**			**Metabolite from *P. rettgeri* (OP830498),),**		
1	7.00	Methyl lactate	1.90	8.02	Methyl lactate	1.20
2	14.23	Cyclohexanol, 5-methyl-2-(1-methylethyl)-	1.86	10.50	Cyclohexanol, 5-methyl-2-(1-methylethyl)-	5.73
3	15.98	Pentanoic acid, 2-methylbutyl ester	1.94	11.43	Pentanoic acid, 2-methylbutyl ester	2.31
4	18.80	9,12-Octadecadienoic acid (Z,Z)-	4.90	13.25	9,12-Octadecadienoic acid (Z,Z)-	6.05
5	20.50	Tetradecanoic acid	8.85	16.50	Tetradecanoic acid	6.26
6	23.11	n-Hexadecanoic acid	2.92	20.81	Dibutyl phthalate	3.61
7	24.06	Dibutyl phthalate	1.55	23.03	9-Octadecenoic acid (Z)-, methyl ester	2.93
8	26.98	3,7,11,15-tetramethyl-2-hexadecen-1-ol	3.83	26.02	3,7,11,15-tetramethyl-2-hexadecen-1-ol	17.54
9	28.15	Octadecane	3.14	28.50	Octadecane	3.99
10	31.50	9-Octadecenoic acid (Z)-, methyl ester	1.90	32.50	n-Hexadecanoic acid	21.96
11	33.98	Phytol	1.96	35.71	Phytol	3.88
12	35.50	Oleic acid	29.23	37.20	Oleic acid	1.63
13	37.00	9,12-Octadecadienoic acid (Z,Z)-	2.62	37.80	9,12-Octadecadienoic acid (Z,Z)-	12.45
14	39.00	11,14,17-Eicosatrienoic acid, methyl ester	25.17	39.75	11,14,17-Eicosatrienoic acid, methyl ester	2.56
15	40.00	Squalene	4.37	40.75	Squalene	3.04
16	48.49	Stigmasterol	1.94	43.52	Stigmasterol	3.27
17	54.00	Lupeol	1.66	48.21	Stearyltrimethylammonium chloride	1.18
	**Metabolite from *Bacillus cereus* (OP830501)**					
1	7.61	Methyl lactate	4.06	2.03		
2	14.13	Cyclohexanol, 5-methyl-2-(1-methylethyl)-	2.13	1.73		
3	15.98	Pentanoic acid, 2-methylbutyl ester	2.67	1.87		
4	18.80	9,12-Octadecadienoic acid (Z,Z)-	4.27	5.83		
5	20.50	Tetradecanoic acid	15.00	16.24		
6	23.11	n-Hexadecanoic acid	3.20	2.51		
7	24.00	Dibutyl phthalate	4.03	2.55		
8	26.98	3,7,11,15-tetramethyl-2-hexadecen-1-ol	4.04	3.29		
9	28.15	Octadecane	2.16	1.82		
10	31.50	9-Octadecenoic acid (Z)-, methyl ester	1.07	1.80		
11	33.98	Phytol	1.25	1.52		
12	35.50	Oleic acid	26.69	27.33		
13	37.00	9,12-Octadecadienoic acid (Z,Z)-	5.03	4.10		
14	39.00	11,14,17-Eicosatrienoic acid, methyl ester	19.22	20.63		
15	40.00	Squalene	3.19	3.00		
16	48.49	Stigmasterol	1.06	1.81		
17	54.00	Lupeol	0.92	1.60		

In addition, the metabolites from *S. liquefaciens* (OP830503) revealed the presence of Tetradecanoic acid (8.85%), Phytol (29.23%) and 11,14,17-Eicosatrienoic acid, methyl ester (25.17%) as the most dominant. In the case of the metabolite from *P. rettgeri* (OP830498), Cyclohexanol, 5-methyl-2-(1-methylethyl)- (5.73%), 9,12-Octadecadienoic acid (Z,Z)- (6.05%), Tetradecanoic acid (6.26%), 3,7,11,15-tetramethyl-2-hexadecen-1-ol (17.54%), n-Hexadecanoic acid (21.96%) and 9,12-Octadecadienoic acid (Z,Z)- (12.45%) were the most dominant ( Table 9).

The metabolites from *B. cereus* (OP830501) were Tetradecanoic acid (15.00%), Oleic acid (26.69%) and 11,14,17-Eicosatrienoic acid, methyl ester (19.22%) as the most dominant compounds ( Table 9).

## Discussion

Seeds that germinate vigorously offers better crop yield. ^38^ For cowpea, generally, final percent germination reached a significantly highest value at shorter steeping duration, then decreased afterwards. However, for Isolate N and O, all values were statistically the same or nearly so. With soybean, it increased with increasing steeping duration until 1, 2, or 3 h, then decreasing afterward, even though this decrease was sometimes insignificant. Although, microbial metabolites are known to promote germination, ^39^^–^^41^ this pattern of result for cowpea and soybean could be ascribed to nutrient and electrolyte leakage at prolonged steeping duration. ^42^ The risk of bacterial and fungal proliferation which can lead to seed decay and spoilage is increased when large endosperm seeds such as soybean and cowpea are primed at longer steeping periods and sown immediately as in this study.

Priming for a shorter period may not allow enough water and bioactive metabolites to enter the seeds and too long a priming period may cause seeds not to germinate. ^43^ The results of this study shows that steeping soybean and cowpea for shorter periods is sufficient for maximal germination values. Steeping duration did not seem to have an impact on final germination pattern on sesame, as high values were distributed throughout without order. There was also no order to the distribution of FPG values for okra, indicating that duration of steeping in the metabolites did not significantly influence germination. This uneven germination pattern could be linked to the hard seed coat of okra, ^44^ which perhaps limited imbibition. This hard seed coat resulted in very high maximal steeping durations of 12 h ^45^ and 48 h ^46^ for okra. Insufficient imbibition can lead to germination delay, ^47^ a situation that could have happened with a hardy seed such as okra in this study, since the highest steeping duration in this study was 5 h and low final germination values were recorded for it.

The effect of steeping duration on mean germination time was negligible for all the crops, as no patterns were observed. All the values obtained “congregated’ around 5. It therefore makes no agronomic sense to attach any importance to significant results here. However, other researchers have reported better mean germination times. ^48^^,^^49^

A higher germination index shows that germinated seeds appeared faster. In the case of cowpea, there was a general decrease in values with increasing steeping time for all isolates and this decline was significant for some isolates. There was also a general but insignificant decline from lower to higher steeping durations with soybean. However, for sesame and okra, steeping time was not significantly affecting the values. Okra required a 48-hour of steeping duration for maximal germination index. ^46^

Vigor index reached its statistical highest at a lower steeping duration for all the isolates in the case of cowpea, then decreased afterwards, and this same pattern was also observed for soybean and sesame. The highest value for okra was not limited to lower steeping periods. In fact, the significantly highest value was obtained at either 3 or 4 h for virtually all isolates. A high steeping duration of 48 hours produced the best vigor index in okra. ^46^ The seed coat of okra limit ample imbibition at short steeping periods.

There was generally no observable pattern for cowpea, soybean, and sesame, and abrupt drop in values were observed for okra with regards to concentration. This may have a lot to do with the erratic emergence of okra seeds ^50^ than any other thing. Similarly, germination was also found to be concentration-independent in the biopriming of canola seeds with varying concentrations of bacterial cell-free supernatants of *Devosia* sp. (SL43). ^51^ While increasing concentration did not impact germination negatively, it is usually not the case with chemical or hormonal priming where the impact of priming on germination and seedling parameters seems to always be concentration-dependent, with negative and drastic effects at higher concentrations. ^52^^,^^53^

Concentration was not effective in producing an impact of significant agronomic proportion on mean germination time in all the crops, as values clustered around 5. With respect to germination index, there was no clear pattern for cowpea, and generally for soybean and okra, too. For metabolites from isolates L, M, and O used in the priming of sesame seeds, there was a rare increase in GIX with increasing concentration, that was also significant.

For cowpea, seedling vigor index gradually peaked at lower concentration for all isolates, then decreased with increasing concentration, although the decreased was statistically insignificant at times. Similarly, for soybean it peaked at a lower concentration, then a significant decline was observed. Sesame recorded no clear pattern with increasing concentration, however, in the case of isolate K, it peaked at a lower concentration, then decreased steadily afterwards, and for isolate L, it rose steadily and peaked significantly at the highest concentration. Mostly, there was no directional change with concentration for seedling vigor index of okra seeds steeped in these metabolites. The hard seed coat of okra is responsible for the ambiguous response of okra to priming at different concentrations.

The GC-MS analysis of the extracts detected the presence of a number metabolites in the metabolome of each of them, some of which were common to all isolates. The compounds detected belong to categories such as alkanes, alcohols, carboxylic acids, esters, and terpenes. The ability of alcohols such as 2,3-butanediol produced by various species of *Bacillus* to promote the growth of *Arabidopsis thaliana* has been reported. ^54^^–^^56^ Tetrahydrofuran-3-ol and 2-heptanone 2-ethyl-1-hexanol from *Bacillus* species as well have also been reported to improve the growth of *A.* *thaliana* and tomato. ^57^

Oleic acid has been commonly detected in the metabolomes of some rhizobacteria. ^58^ It was detected in all five strains in this study. n-Hexadecanoic acid, a metabolite detected in the metabolome of two of the isolates in this study, and hexadecane, have been shown to possess the ability to improve the growth of *Vigna radiata.* ^59^

## Conclusion

This study enables us to understand the dynamics surrounding the effectiveness of microbial metabolites, something that is not possible with typical screening assays that employs a constant concentration. However, the function of the various metabolites in the isolates sampled needs to be properly investigated to identify the bioactive metabolites responsible for growth promotion. The organisms themselves will have to be further studied to better understand the production of the bioactive metabolites.

## Data Availability

Figshare. Raw data on germinability parameters at different metabolite concentrations and steeping duration in microbial metabolite. DOI: https://doi.org/10.6084/m9.figshare.23284865.v1. ^60^
