# Effects of steeping duration and concentration of metabolites from rhizosphere bacteria on the germinability of cowpea, soybean, sesame, and okra seeds

**DOI:** 10.12688/f1000research.137322.2

**Published:** 2024-01-09

**Authors:** Oghenerobor Akpor, Ayotunde Ajinde, Tolulope Ogunnusi

**Affiliations:** 1 Department of Biological Sciences, Afe Babalola University, Ado Ekiti, Ekiti, 360102, Nigeria

**Keywords:** Germinability, rhizobacteria, germinability, seed vigor

## Abstract

Seed priming enhances germination and growth, which are important determinants of crop yield. This study was carried out to assess the effect of steeping duration and metabolite concentration on the priming of five (5) different crops, using the metabolites of five (5) bacterial isolates. The crop seeds were treated in the cold-extracted metabolites of the five isolates at five (5) different steeping durations (1, 2, 3, 4, and 5 h) and then in five metabolite concentrations (200, 400, 600, 800, and 1000 mg/L) of the five extracted metabolites at the optimal steeping duration determined in the first experiment. Characterization of the cold-extracted metabolites was also carried out using gas-chromatography-mass spectrometry (GC-MS). Results revealed that steeping cowpea and soybean for longer durations (< 3 h) could hinder their growth and development. Lower concentrations were observed to be optimal for cowpea and soybean, but for sesame and okra, there was no detectable pattern with metabolite concentration. The GC-MS revealed the presence of some molecules (e.g. hexadecanoic acid) that have shown plant growth promotion potential in other studies. This study showed that seeds with large endosperm, such as, cowpea and soybean, are more prone to the deleterious effects of treatment for longer durations. Further experiments should be carried out to isolate and purify the bioactive moieties for further studies and onward application.

## Introduction

Seeds are critical inputs in agricultural production. Hence, it is expedient to plant only seeds that can produce the best agronomic performance. To this end, seed priming has been proposed and used. The beneficial effects of seed priming on a variety of crops have been confirmed. ^1– 4^ Although priming, as a plant-growth enhancement technique, can be applied at many stages in the developmental cycle of a plant, seed treatment is popular due to its practicality and simplicity.

Priming usually involves soaking seeds in a solution to kickstart various pre-germinative activities, ^5^^,^^6^ and it typically requires re-drying the seed before planting. ^7^ Several priming methods are used to enhance the agro-morphic parameters of crop seeds. Hydropriming involves steeping seeds in water ^8^; osmopriming entails the use of an osmoticum ^9^; halopriming involves soaking in salt solutions ^10^; solid-matrix priming involves priming on a solid material ^11^; and hormonal priming, which requires the use of plant growth regulators such as abscisic acid (ABA), ^12^^,^^13^ gibberellic acids (GAs) ^5^ or salicylic acid (SA). ^14^^–^^16^ Biopriming, which involves using microbial products, is a relatively new priming strategy that offers the advantage of environmental friendliness and may also be less expensive than most of the priming methods available.

Seed priming can enhance germination, reduce germination time, and improve seedling vigor. ^17^ Priming also increases the resistance of seeds to environmental stress. ^18^ Although the exact mechanism of priming is not well understood, it is understood to involve specific physiological and biochemical reactions. ^19^^,^^20^

Optimal priming duration is plant-specific due to seed structures that are unique morphologically and physiologically. Since bacteria secret substances that can promote plant growth, ^21^^,^^22^ the determination of their effective concentration is necessary for wholesome application. Optimal concentrations of chemicals used in priming have been determined. ^23^^–^^25^

However, while there is ample research on other priming methods, microbial priming is less well-researched, and their optimization is even less so. Hence, we set out to understand the impact of steeping duration and metabolite concentration on the growth promotion activity of secondary metabolites on selected crops (cowpea, soybean, sesame, and okra) vis-a-vis some agro-morphic parameters by sowing seeds soaked in metabolites of some previously-isolated bacteria immediately without drying. The crops used for priming in this study are of significant economic importance in tropical regions where they are a vital source of dietary requirements. Therefore, it is necessary to boost their production, and seed priming is a veritable tool for achieving this end.

## Methods

### Isolation

Seventy-five (75) bacterial strains were isolated from rhizospheres within Afe Babalola University (Ado-Ekiti, Nigeria) using the pour-plate technique, as reported by Sanders. ^26^ The five (5) strains used in this study were chosen based on their germinability enhancement potential observed from a previous *in planta* experiment using seeds (data not presented). Pure cultures of the five (5) strains were stored on nutrient agar slants at 4°C ± 2°C until needed.

### Molecular identification

Identification of the 15 rhizobacterial strains was done using the 16s rRNA gene sequencing technique. The CTAB protocol was used in DNA extraction. ^27^ Subsequently, the V3-V4 hypervariable regions of the 16s rRNA gene were amplified using 27F 5′ AGAGTTTGATCMTGGCTCAG 3′ and 1525R 5′AAGGAGGTGATCCAGCC 3′ primers and the PCR conditions (profile) comprised an initial denaturation at 94°C for 5 min; followed by 30 cycles consisting of 94°C for 30 s, annealing at 50°C for 60 s, and extension at 72°C for 1 min 30 s; and a final extension at 72°C for 10 mins, in a GeneAmp 9700 PCR System Thermalcycler (Applied Biosystem Inc., USA). The integrity check of the amplified products was carried out using 1% Agarose gel and purified using 95% ethanol and 3M of sodium acetate. The purified PCR products were sequenced using a Genetic Analyzer 3130xl sequencer (Applied Biosystem Inc., USA) according to the manufacturer’s instructions. The sequences were compared to those in a database for identification via the NCBI site (BLAST). Then, the 16S rRNA sequences were aligned using the ClustalW program, and the neighbor-joining phylogenetic tree was constructed using MEGA 11. ^28^ The isolates were identified as OP830504 – *Serratia liquefaciens* AYO-O; OP830503 – *S. liquefaciens* AYO-N; OP830491 – *Providencia rettgeri* AYO-B; OP830498 – *P. rettgeri* AYO-I; and OP830501 – *Bacillus cereus* AYO-L.

### Test seeds

The seeds used for the study were cowpea ( *Vigna unguiculata*), soybean ( *Glycine max*), sorghum ( *Sorghum bicolor*), sesame ( *Sesamum indicum*), and okra ( *Abelmoschus esculentus*). All the seeds were sourced from a local seed dealer in Ado-Ekiti (Ekiti State, Nigeria).

First, a test was carried out on seeds in order to assess their viability. For this, over (500) seeds were released into a beaker containing 500 mL of sterile, distilled water, and floated (unviable) seeds were collected and discarded, and the water decanted. From the remaining seeds, 50 seeds were sown in four replicates on 3.5 grams of absorbent cotton wool placed within transparent plastic containers measuring 100 cm by 40 cm. Incubation was under fluorescent light with daily watering for five (5) days under laboratory conditions. A final germination percentage of 80% was indicative of good germination potential (seed quality) for all the seeds, except okra at 60%.

### Test metabolites

The metabolites were extracted from the cultures of the five strains: A = OP830504 – *Serratia liquefaciens* AYO-O; B = OP830503 – *S. liquefaciens* AYO-N; C = OP830491 – *Providencia rettgeri* AYO-B; D = OP830498 – *P. rettgeri* AYO-I; and E = OP830501 – *Bacillus cereus* AYO-L.

The cold extraction method reported by Ref. 29 was adopted. The 48-hour broth cultures of the isolates grown for 48 h at 25°C ± 2°C were centrifuged at 5000 rpm for 15 min to obtain the cell-free supernatants, which were acidified to a pH of 2 by the addition of 1M HCl. Following acidification, an equal volume of methanol:ethylacetate (2:1) mixture was added and incubated at 4°C ± 2°C for 24 h. After incubation, the mixtures were transferred to a separating funnel to separate the solvent from the broth and precipitate the metabolite. The precipitated metabolite was then dried by placing the beakers containing the separated solvents in a water bath at a temperature of 80°C. The dried metabolite was then quantified and stored in clean universal bottles at 4°C ± 2°C until needed.

### Metabolite characterization

Metabolite identification was carried out using a gas chromatograph (GC) connected to a mass spectrometer (Varian 3800/4000; Agilent Technologies, USA). The equipment has a splitter split/splitless HP5 (30 m × 0.25 mm) silica-based, cross-linked column with nitrogen as a gas carrier. The injector and detector temperatures were set at 300°C. The GC temperature regimen started at 50°C and increased to 100°C with a ramp rate of 10°C min-1 and held at 100°C for 2 minutes and was increased to 250°C at the rate of 5 min ^-1^ for 2 mins. It was finally raised to 300°C with a ramp rate of 3°C min ^−1^ for 15 minutes. A sample volume of 1 uL was used and the carrier gas at a rate of 1 mL min ^−1^. The MS was scanned from 30–400 amu at 1.562 u s ^−1^ and operated in EI mode at 70 eV. The mass spectral data were compared with those of the National Institute of Standards and Technology (NIST) and Wiley libraries. Only the mass spectral data of compounds with at least 90% matching accuracy were reported.

### Germinability studies

Germinability experiments were carried out to investigate the effects of steeping duration and metabolite concentration on the seeds.

The investigation of the effect of steeping duration on the germinability of the seeds was carried out for 1, 2, 3, 4, or 5 h. Viable, surface-sterilized seeds of the four seeds were treated in a 1000 mg/L (water dilution) of the five metabolites. Every one hour, for a 5-h duration, seven (7) seeds were withdrawn and planted in transparent plastic cups in six (6) replicates and incubated for 8 days with daily watering. At the expiration of incubation, final germination percentage, mean germination time, germination index and vigor index were estimated as follows:

• Final germination percentage (FGP) = total number of germinated seeds/total number of seeds sown×100% ^30^
• Mean germination time (MGT) = ∑fxf ^31^
  Where f is the number of seeds germinated on day x
• Germination index (GIX) = 8×N1+7×N2+6×N3+ … +1×N8 ^32^
  Where N1, N2, N3 … N8 represent the number of seeds that germinated on the first, second, and third until the 8th day, and 8, 9, 7 … 1 are the weights given to the number of germinated seeds on the first, second, and third day up to the 8th day.
• Vigor index (VIX) = FGP × average plant height ^33^

For effect of metabolite concentration, 200 mg/L, 400 mg/L, 600 mg/L, 800 mg/L, and 1000 mg/L (water dilutions) were used for the study. The seeds were treated in the respective metabolite concentrations and allowed to stand for the optimal steeping time obtained in the first experiment before planting and incubation using six (6) replicates. At the expiration of the 8-day incubation period, final germination percentage, mean germination time, germination index, and vigor index were estimated as described earlier.

## Results

### Effect of steeping duration in metabolites

Generally, the final germination percentage of the cowpea seeds showed significantly highest and lowest values in seeds treated for 2 and 5 h, 1 and 5 h, 1, 2, and 3 and 4 h in the metabolites from Isolates K, L and M, respectively. There was no significant difference in final germination percentage at the different steeping durations for seeds treated in the metabolite from Isolate N. In addition, seeds treated with the metabolite from Isolate O showed significantly lowest final germination percentage at 3 and 5 h. With respect to mean germination time, significantly lowest values were recorded for seeds steeped for 1 h (metabolites from Isolates K and M), 4 and 5 h (metabolite from isolate L), 1, 4, and 5 h (metabolites from Isolate N), and 1, 2, and 4 (metabolite from Isolate O). Germination index showed significantly lowest values at 3 h (metabolite from Isolate L), 4 and 5 h (metabolite from Isolate M), 1-3 h (metabolite from Isolate N), and 3 and 5 h (metabolite from Isolate O). For vigor index, significantly lowest values were observed at 2, 4 and 5 h (metabolite from Isolate M), 1, 3, and 5 h (metabolite from Isolate N), and 4 and 5 h (metabolite from Isolate O). Also, germination and vigor indices of the seeds showed significantly lowest values in setups treated for 5 h (metabolites K and L) ( Table 1).

**Table 1.** Germinability of the cowpea seeds at the different steeping durations in the respective metabolites.

Parameter	Time	Isolate K	Isolate L	Isolate M	Isolate N	Isolate O
FPG	1 h	71.43 ^a^ (±0.00)	78.57 ^a^ (±7.82)	85.71 ^a^ (±0.00)	64.29 ^a^ (±7.82)	78.57 ^a^ (±7.82)
	2 h	85.71 ^b^ (±15.65)	64.29 ^b^ (±7.82)	71.43 ^ac^ (±15.65)	78.57 ^a^ (±7.82)	78.57 ^a^ (±7.82)
	3 h	71.43 ^a^ (±0.00)	42.86 ^c^ (±15.65)	71.43 ^ac^ (±31.30)	71.43 ^a^ (±0.00)	50.00 ^b^ (±23.47)
	4 h	71.43 ^a^ (±0.00)	57.14 ^b^ (±0.00)	50.00 ^b^ (±7.82)	78.57 ^a^ (±23.47)	78.57 ^a^ (±7.82)
	5 h	54.29 ^c^ (±15.65)	21.43 ^d^ (±7.82)	64.29 ^bc^ (±7.82)	78.57 ^a^ (±7.82)	64.29 ^ab^ (±23.47)
MGT	1 h	5.14 ^a^ (±0.05)	5.45 ^a^ (±0.25)	5.04 ^a^ (±0.04)	5.14 ^a^ (±0.06)	5.24 ^a^ (±0.06)
	2 h	5.51 ^b^ (±0.15)	5.37 ^ab^ (±0.01)	5.34 ^b^ (±0.02)	5.36 ^b^ (±0.08)	5.25 ^a^ (±0.17)
	3 h	5.32 ^c^ (±0.04)	5.23 ^b^ (±0.00)	5.39 ^b^ (±0.06)	5.40 ^b^ (±0.17)	5.40 ^b^ (±0.16)
	4 h	5.43 ^b^ (±0.08)	5.11 ^c^ (±0.00)	5.41 ^b^ (±0.10)	5.21 ^a^ (±0.11)	5.25 ^a^ (±0.07)
	5 h	5.38 ^bc^ (±0.08)	5.25 ^bc^ (±0.27)	5.57 ^c^ (±0.07)	5.24 ^a^ (±0.06)	5.45 ^b^ (±0.15)
GIX	1 h	129.50 ^a^ (±3.83)	120.00 ^a^ (±29.58)	164.50 ^a^ (±3.83)	116.00 ^ab^ (±18.62)	133.50 ^a^ (±8.22)
	2 h	125.00 ^a^ (±33.96)	101.50 ^a^ (±11.50)	115.50 ^b^ (±26.84)	124.00 ^ab^ (±18.62)	133.00 ^a^ (±0.00)
	3 h	116.00 ^a^ (±3.29)	73.50 ^b^ (±26.84)	112.50 ^b^ (±53.13)	110.00 ^b^ (±10.95)	79.00 ^b^ (±44.91)
	4 h	109.00 ^a^ (±4.38)	105.00 ^a^ (±0.00)	77.00 ^c^ (±7.67)	133.50 ^a^ (±31.22)	131.00 ^a^ (±9.86)
	5 h	84.40 ^b^ (±19.72)	35.00 ^c^ (±7.67)	89.50 ^bc^ (±16.98)	133.50 ^a^ (±8.22)	93.00 ^b^ (±25.20)
VIX	1 h	818.88 ^a^ (±69.86)	862.35 ^a^ (±163.31)	1578.37 ^a^ (±104.63)	783.78 ^a^ (±215.62)	943.06 ^ab^ (±2.46)
	2 h	1212.14 ^b^ (±240.22)	575.61 ^b^ (±130.67)	697.76 ^bc^ (±202.77)	1390.10 ^b^ (±258.55)	1064.49 ^a^ (±77.35)
	3 h	788.78 ^a^ (±32.42)	323.67 ^c^ (±239.21)	888.57 ^c^ (±625.97)	700.51 ^a^ (±100.04)	608.47 ^b^ (±535.09)
	4 h	877.55 ^a^ (±125.19)	507.35 ^b^ (±66.62)	343.98 ^b^ (±156.16)	1305.20 ^b^ (±800.23)	556.43 ^c^ (±266.37)
	5 h	434.12 ^c^ (±247.37)	37.55 ^d^ (±29.51)	353.88 ^b^ (±68.41)	999.18 ^ab^ (±229.37)	466.53 ^c^ (±347.41)

Values are averages of six replicates. Values in parenthesis represent ± standard deviations of means. Same and different superscripts on same column for a particular factor connote no significant difference and significant difference, respectively. K, L, M, N, and O represent *Serratia liquefaciens,* (OP830504), *S. liquefaciens* (OP830503), *Providencia rettgeri* (OP830491), *P. rettgeri* (OP830498), and *Bacillus cereus* (OP830501). FPG = final percent germination, MGT = mean germination time, GIX = germination index, and VIX = vigor index.

In the case of the soybean seeds, the final germination percentage of the seeds showed the highest values at 2 h steeping duration when treated in the respective metabolites, apart from those treated in Isolate O, where 1 h steeping duration was observed to show the highest value. Furthermore, significantly lowest mean germination times were recorded in seeds steeped for 1, 2, and 3 h, 1 and 2 h, 1, 4, and 5 h in the metabolites from isolates L, M and N, respectively, and 2 h in the metabolites from Isolate K, and 1, 3, and 4 h in the metabolite from Isolate O. However, significantly highest germination index was observed in the seeds treated for 1 and 2 h in Metabolite K, 2 h in Metabolite L, 1-3 h in Metabolite M, 2 and 3 h in Metabolite N, and 1 h in Metabolite O. Also, for seedling vigor index, significantly highest values were observed at 2 h (Metabolites K and L), 1, 3, 4, and 5 h (Metabolite M), 2 and 3 h (Metabolite N), and 1 h (Metabolite O) ( Table 2).

**Table 2.** Germinability of the soybean seeds at the different steeping durations in the respective metabolites.

Parameter	Time	Isolate K	Isolate L	Isolate M	Isolate N	Isolate O
FPG	1 h	64.29 ^a^ (±7.82)	50.00 ^ad^ (±7.82)	57.14 ^ac^ (±15.65)	50.00 ^a^ (±7.82)	85.71 ^a^ (±0.00)
	2 h	71.43 ^a^ (±15.65)	64.29 ^b^ (±7.82)	71.43 ^b^ (±0.00)	78.57 ^b^ (±7.82)	57.14 ^bc^ (±0.00)
	3 h	42.86 ^b^ (±15.65)	42.86 ^a^ (±0.00)	71.43 ^b^ (±0.00)	71.43 ^b^ (±15.65)	50.00 ^b^ (±7.82)
	4 h	57.14 ^ab^ (±15.65)	57.14 ^bd^ (±0.00)	50.00 ^a^ (±7.82)	57.14 ^a^ (±0.00)	50.00 ^b^ (±7.82)
	5 h	57.14 ^ab^ (±0.00)	50.00 ^ad^ (±7.82)	64.29 ^bc^ (±7.82)	50.00 ^a^ (±7.82)	64.29 ^c^ (±7.82)
MGT	1 h	5.37 ^a^ (±0.01)	5.34 ^a^ (±0.02)	5.31 ^a^ (±0.14)	5.50 ^a^ (±0.25)	5.51 ^ab^ (±0.21)
	2 h	5.27 ^b^ (±0.05)	5.44 ^ac^ (±0.06)	5.37 ^a^ (±0.10)	5.69 ^b^ (±0.10)	5.61 ^a^ (±0.12)
	3 h	5.67 ^c^ (±0.06)	5.41 ^ac^ (±0.10)	5.53 ^b^ (±0.15)	5.72 ^b^ (±0.15)	5.34 ^b^ (±0.12)
	4 h	5.48 ^d^ (±0.02)	5.65 ^b^ (±0.04)	5.55 ^b^ (±0.06)	5.61 ^ab^ (±0.12)	5.52 ^ab^ (±0.23)
	5 h	5.50 ^e^ (±0.00)	5.50 ^c^ (±0.20)	5.60 ^b^ (±0.01)	5.50 ^a^ (±0.00)	5.54 ^a^ (±0.08)
GIX	1 h	101.50 ^ab^ (±11.50)	80.50 ^a^ (±11.50)	95.00 ^ac^ (±33.96)	71.50 ^a^ (±0.55)	124.50 ^a^ (±16.98)
	2 h	119.00 ^b^ (±23.00)	98.00 ^b^ (±15.34)	112.50 ^a^ (±7.12)	99.00 ^b^ (±4.38)	78.00 ^bc^ (±6.57)
	3 h	57.00 ^c^ (±23.00)	66.50 ^c^ (±3.83)	101.00 ^ac^ (±12.05)	91.00 ^bc^ (±27.39)	81.00 ^bd^ (±18.62)
	4 h	84.50 ^a^ (±23.55)	75.50 ^ac^ (±2.74)	70.50 ^b^ (±8.22)	78.00 ^ac^ (±6.57)	71.00 ^b^ (±1.10)
	5 h	84.00 ^a^ (±0.00)	71.50 ^ac^ (±1.64)	88.50 ^c^ (±11.50)	73.50 ^a^ (±11.50)	92.00 ^cd^ (±15.34)
VIX	1 h	490.00 ^a^ (±60.14)	326.63 ^a^ (±28.50)	607.45 ^a^ (±339.48)	241.73 ^ad^ (±85.74)	1027.96 ^a^ (±329.30)
	2 h	926.73 ^b^ (±416.05)	593.67 ^b^ (±120.95)	845.41 ^b^ (±39.68)	897.14 ^b^ (±367.98)	494.69 ^b^ (±66.17)
	3 h	243.67 ^a^ (±223.11)	252.24 ^a^ (±91.88)	805.10 ^ab^ (±60.36)	711.63 ^bc^ (±437.06)	418.88 ^b^ (±196.62)
	4 h	474.59 ^a^ (±374.35)	305.71 ^a^ (±75.56)	422.45 ^a^ (±135.48)	556.73 ^c^ (±15.20)	243.67 ^c^ (±44.26)
	5 h	346.94 ^a^ (±76.01)	279.08 ^a^ (±85.06)	440.00 ^a^ (±110.89)	232.96 ^d^ (±115.02)	494.59 ^b^ (±132.24)

Values are averages of six replicates. Values in parenthesis represent ± standard deviations of means. Same and different superscripts on same column for a particular factor connote no significant difference and significant difference, respectively. K, L, M, N, and O represent *Serratia liquefaciens,* (OP830504), *S. liquefaciens* (OP830503), *Providencia rettgeri* (OP830491), *P. rettgeri* (OP830498), and *Bacillus cereus* (OP830501). FPG = final percent germination, MGT = mean germination time, GIX = germination index, and VIX = vigor index.

For the sesame seeds, remarkably high final germination values (> 78%) were observed in the respective treatments, regardless of the steeping duration. However, a significantly lowest mean germination time was observed for seeds treated for 1 h (metabolites from Isolate O), 2 h (metabolites from Isolates L and M) and 1, 2, 3, and 5 h (metabolites from Isolate K). Also, significantly highest germination index was observed for seeds treated for 2 and 4 h (metabolite from Isolate K), 2 h (metabolites from Isolates L and M) and 1 h (metabolites from Isolates N and O). In the case of the seedling vigor index, seeds treated for 2 and 3 h (metabolites from Isolates K and O), 1 h (metabolite from Isolates L and M) and 3 h (metabolite from Isolate N) showed the significantly highest values ( Table 3).

**Table 3.** Germinability of the sesame seeds at the different steeping durations in the respective metabolites.

Parameter	Time	Isolate K	Isolate L	Isolate M	Isolate N	Isolate O
FPG	1 h	92.86 ^a^ (±7.82)	100.00 ^a^ (±0.00)	100.00 ^a^ (±0.00)	92.86 ^a^ (±7.82)	100.00 ^a^ (±0.00)
	2 h	100.00 ^a^ (±0.00)	100.00 ^a^ (±0.00)	100.00 ^a^ (±0.00)	100.00 ^b^ (±0.00)	100.00 ^a^ (±0.00)
	3 h	92.86 ^a^ (±7.82)	85.71 ^b^ (±0.00)	92.86 ^b^ (±7.82)	100.00 ^b^ (±0.00)	92.86 ^b^ (±7.82)
	4 h	100.00 ^a^ (±0.00)	78.57 ^c^ (±7.82)	100.00 ^a^ (±0.00)	100.00 ^b^ (±0.00)	92.86 ^b^ (±7.82)
	5 h	92.86 ^a^ (±7.82)	100.00 ^a^ (±0.00)	92.86 ^b^ (±7.82)	100.00 ^b^ (±0.00)	100.00 ^a^ (±0.00)
MGT	1 h	5.33 ^ab^ (±0.14)	5.42 ^a^ (±0.00)	5.30 ^a^ (±0.04)	5.00 ^a^ (±0.00)	5.16 ^a^ (±0.04)
	2 h	5.27 ^ab^ (±0.08)	5.20 ^b^ (±0.00)	5.13 ^b^ (±0.15)	5.42 ^a^ (±0.00)	5.38 ^b^ (±0.04)
	3 h	5.25 ^a^ (±0.02)	5.27 ^c^ (±0.05)	5.37 ^a^ (±0.06)	5.34 ^a^ (±0.00)	5.37 ^b^ (±0.04)
	4 h	5.34 ^b^ (±0.00)	5.35 ^d^ (±0.04)	5.34 ^a^ (±0.08)	5.34 ^a^ (±0.00)	5.53 ^c^ (±0.03)
	5 h	5.33 ^ab^ (±0.01)	5.34 ^d^ (±0.08)	5.50 ^c^ (±0.00)	5.42 ^a^ (±0.00)	5.38 ^b^ (±0.04)
GIX	1 h	148.50 ^a^ (±0.55)	154.00 ^a^ (±0.00)	164.50 ^a^ (±3.83)	182.00 ^a^ (±15.34)	178.50 ^a^ (±3.83)
	2 h	168.00 ^b^ (±7.67)	175.00 ^b^ (±0.00)	182.00 ^b^ (±15.34)	154.00 ^b^ (±0.00)	157.50 ^b^ (±3.83)
	3 h	157.50 ^a^ (±11.50)	143.50 ^c^ (±3.83)	147.00 ^c^ (±7.67)	161.00 ^b^ (±0.00)	147.00 ^c^ (±15.34)
	4 h	161.00 ^b^ (±0.00)	126.00 ^d^ (±15.34)	161.00 ^a^ (±7.67)	161.00 ^b^ (±0.00)	133.50 ^d^ (±8.22)
	5 h	150.50 ^a^ (±11.50)	161.00 ^a^ (±7.67)	136.50 ^c^ (±11.50)	154.00 ^b^ (±0.00)	157.50 ^b^ (±3.83)
VIX	1 h	528.67 ^a^ (±111.00)	800.00 ^a^ (±178.40)	952.86 ^a^ (±7.82)	666.53 ^a^ (±93.00)	756.43 ^a^ (±71.20)
	2 h	711.43 ^b^ (±12.52)	660.00 ^b^ (±115.80)	887.86 ^a^ (±2.35)	506.43 ^bd^ (±44.60)	877.86 ^b^ (±28.95)
	3 h	678.16 ^b^ (±33.31)	615.31 ^b^ (±2.01)	721.63 ^b^ (±42.48)	829.29 ^c^ (±97.81)	791.43 ^ab^ (±78.25)
	4 h	592.86 ^a^ (±31.30)	339.18 ^c^ (±22.80)	582.86 ^c^ (±50.08)	553.57 ^b^ (±44.60)	447.55 ^c^ (±138.83)
	5 h	511.73 ^a^ (±95.12)	455.71 ^d^ (±0.00)	461.73 ^d^ (±106.08)	450.71 ^d^ (±22.69)	496.43 ^c^ (±44.60)

Values are averages of six replicates. Values in parenthesis represent ± standard deviations of means. Same and different superscripts on same column for a particular factor connote no significant difference and significant difference, respectively. K, L, M, N, and O represent *Serratia liquefaciens,* (OP830504), *S. liquefaciens* (OP830503), *Providencia rettgeri* (OP830491), *P. rettgeri* (OP830498), and *Bacillus cereus* (OP830501). FPG = final percent germination, MGT = mean germination time, GIX = germination index, and VIX = vigor index.

When the okra seeds were treated in the different metabolites for varying durations, final germination percentage of the okra seeds showed significantly highest values in seeds treated for 3 h (Metabolite K), 4 h (Metabolite L) and 1 and 5 h (Metabolite N). There was no significant difference in final germination percentage between seeds treated with the metabolite from Isolate M at the different steeping durations. Also, mean germination time showed no significant difference at the different steeping durations for seeds treated with the metabolites from Isolates K, M and O. However, significantly lowest mean germination times were observed for seeds treated for 2 and 5 h and 3-5 h with the metabolites from Isolates L and N, respectively. Furthermore, seeds steeped for 3 h showed significantly highest germination and vigor indices when treated with the metabolites from Isolates K respectively. Significantly highest germination index values were observed for seeds treated for 3 h (Metabolite K), 2-4 h (metabolite L), 1, 3, and 4 h (metabolite M), 1, 3, 4, and 5 h (Isolate N), and 1-4 h (Isolate O). Finally, for seedling vigor index, significantly highest values were observed at 3 h (Metabolite K), 3 and 4 h (Metabolite L), 3 and 4 h (Metabolite M), 1, 4, and 5 (Metabolite N), and 3 and 4 h (Metabolite O) ( Table 4).

**Table 4.** Germinability of the okra seeds at the different steeping durations in the respective metabolites.

Parameter	Time	Isolate K	Isolate L	Isolate M	Isolate N	Isolate O
FPG	1 h	28.57 ^a^ (±15.65)	57.14 ^ad^ (±15.65)	85.71 ^a^ (±0.00)	85.71 ^a^ (±0.00)	71.43 ^ab^ (±15.65)
	2 h	71.43 ^b^ (±0.00)	71.43 ^ab^ (±0.00)	78.57 ^a^ (±7.82)	64.29 ^b^ (±7.82)	71.43 ^ab^ (±15.65)
	3 h	85.71 ^c^ (±0.00)	85.71 ^bc^ (±0.00)	78.57 ^a^ (±7.82)	71.43 ^bc^ (±0.00)	85.71 ^a^ (±15.65)
	4 h	64.29 ^b^ (±7.82)	92.86 ^c^ (±7.82)	78.57 ^a^ (±7.82)	64.29 ^b^ (±23.47)	78.57 ^ab^ (±7.82)
	5 h	71.43 ^b^ (±15.65)	50.00 ^d^ (±23.47)	78.57 ^a^ (±23.47)	78.57 ^ac^ (±7.82)	64.29 ^b^ (±7.82)
MGT	1 h	5.41 ^a^ (±0.10)	5.28 ^ac^ (±0.01)	5.30 ^a^ (±0.08)	5.47 ^a^ (±0.20)	5.22 ^a^ (±0.24)
	2 h	5.33 ^a^ (±0.16)	5.14 ^bd^ (±0.05)	5.39 ^a^ (±0.33)	5.91 ^b^ (±0.06)	5.29 ^a^ (±0.23)
	3 h	5.29 ^a^ (±0.07)	5.36 ^a^ (±0.02)	5.30 ^a^ (±0.13)	5.35 ^c^ (±0.04)	5.28 ^a^ (±0.30)
	4 h	5.33 ^a^ (±0.11)	5.39 ^a^ (±0.06)	5.19 ^a^ (±0.01)	5.27 ^c^ (±0.05)	5.20 ^a^ (±0.05)
	5 h	5.28 ^a^ (±0.31)	5.19 ^cd^ (±0.21)	5.38 ^a^ (±0.17)	5.34 ^c^ (±0.02)	5.35 ^a^ (±0.26)
GIX	1 h	45.50 ^a^ (±26.84)	93.00 ^a^ (±24.10)	140.50 ^a^ (±7.12)	124.00 ^a^ (±18.62)	120.00 ^ab^ (±8.76)
	2 h	114.00 ^b^ (±13.15)	129.50 ^b^ (±3.83)	121.00 ^bc^ (±13.15)	71.00 ^b^ (±5.48)	122.50 ^ab^ (±42.17)
	3 h	141.00 ^c^ (±6.57)	133.00 ^b^ (±1.10)	127.50 ^abd^ (±1.64)	113.00 ^a^ (±1.10)	139.00 ^ab^ (±1.10)
	4 h	102.50 ^b^ (±4.93)	144.50 ^b^ (±18.07)	137.00 ^ab^ (±12.05)	108.50 ^a^ (±42.17)	137.00 ^a^ (±18.62)
	5 h	114.50 ^b^ (±2.74)	84.00 ^a^ (±30.67)	120.00 ^cd^ (±24.10)	126.50 ^a^ (±14.79)	102.00 ^b^ (±3.29)
VIX	1 h	108.27 ^a^ (±92.67)	335.31 ^a^ (±154.70)	580.41 ^ac^ (±45.61)	743.27 ^a^ (±73.77)	488.98 ^ac^ (±140.40)
	2 h	495.92 ^bd^ (±36.89)	557.65 ^b^ (±73.22)	539.29 ^ad^ (±126.87)	303.88 ^b^ (±9.17)	522.65 ^ac^ (±230.94)
	3 h	869.39 ^c^ (±8.05)	836.33 ^c^ (±12.07)	643.88 ^ab^ (±133.02)	511.22 ^c^ (±4.47)	818.88 ^b^ (±292.31)
	4 h	537.45 ^b^ (±112.12)	754.08 ^c^ (±109.77)	713.67 ^b^ (±61.93)	553.47 ^ac^ (±336.68)	710.20 ^ab^ (±140.84)
	5 h	410.82 ^d^ (±171.02)	250.41 ^a^ (±222.00)	488.47 ^cd^ (±104.96)	559.90 ^ac^ (±100.27)	392.55 ^c^ (±100.94)

Values are averages of six replicates. Values in parenthesis represent ± standard deviations of means. Same and different superscripts on same column for a particular factor connote no significant difference and significant difference, respectively. K, L, M, N, and O represent *Serratia liquefaciens,* (OP830504), *S. liquefaciens* (OP830503), *Providencia rettgeri* (OP830491), *P. rettgeri* (OP830498), and *Bacillus cereus* (OP830501). FPG = final percent germination, MGT = mean germination time, GIX = germination index, and VIX = vigor index.

### Effect of metabolite concentration

At the respective concentrations of the different metabolites, significantly lowest final germination percentages were observed in the cowpea seeds treated with 800 mg/L of the metabolites from Isolates K, L, and N. The final germination percentage of the cowpea seeds treated with metabolites from isolates M and O did not differ significantly at the respective concentrations. Also, significantly lowest mean germination times were observed for seeds treated with metabolite concentrations of 200, 600, and 800 mg/L (metabolite from Isolate K), 400 and 800 mg/L (metabolite from Isolate L), and 400 mg/L (metabolite from Isolate M), 200 – 800 mg/L (Metabolite N), and 200-600 mg/L (Metabolite O). Significantly highest germination index values were observed in the seeds treated with metabolite concentrations of 200 and 600 mg/L (Metabolite K), 600 mg/L (Metabolite L), 200, 400, 600, and 1000 mg/L (Metabolite N), and 200, 600, and 800 mg/L (Metabolite O) ( Table 5). In the case of seedling vigor index, significantly highest values were observed at concentrations of 600 mg/L (Metabolite K), 200, 600, and 1000 mg/L (Metabolite L), 600 and 1000 mg/L (Metabolite M), 400, 600, and 1000 mg/L (Metabolite N), and 200-600 mg/L (Metabolite O) ( Table 5).

**Table 5.** Germinability of the cowpea seeds at the different concentrations of the respective metabolites.

Parameter	Concentration	Isolate K	Isolate L	Isolate M	Isolate N	Isolate O
FPG	200 mg/L	71.43 ^ab^ (±0.00)	64.29 ^a^ (±23.47)	50.00 ^a^ (±7.82)	64.29 ^a^ (±23.47)	64.29 ^a^ (±7.82)
	400 mg/L	57.14 ^ac^ (±31.30)	42.86 ^b^ (±0.00)	42.86 ^a^ (±0.00)	85.71 ^b^ (±0.00)	57.14 ^a^ (±46.95)
	600 mg/L	85.71 ^b^ (±0.00)	85.71 ^c^ (±0.00)	57.14 ^a^ (±31.30)	85.71 ^b^ (±15.65)	78.57 ^a^ (±7.82)
	800 mg/L	50.00 ^c^ (±7.82)	14.29 ^d^ (±0.00)	50.00 ^a^ (±7.82)	42.86 ^a^ (±0.00)	64.29 ^a^ (±7.82)
	1000 mg/L	71.43 ^abc^ (±15.65)	64.29 ^a^ (±23.47)	57.14 ^a^ (±0.00)	78.57 ^ab^ (±23.47)	71.43 ^a^ (±15.65)
MGT	200 mg/L	5.32 ^a^ (±0.25)	5.67 ^a^ (±0.09)	5.61 ^a^ (±0.12)	5.45 ^a^ (±0.05)	5.37 ^ab^ (±0.01)
	400 mg/L	5.62 ^b^ (±0.12)	5.38 ^bc^ (±0.07)	5.25 ^b^ (±0.27)	5.47 ^ab^ (±0.11)	5.28 ^a^ (±0.31)
	600 mg/L	5.39 ^ab^ (±0.09)	5.47 ^b^ (±0.08)	5.54 ^a^ (±0.04)	5.47 ^ab^ (±0.05)	5.40 ^ac^ (±0.24)
	800 mg/L	5.52 ^ab^ (±0.23)	5.25 ^c^ (±0.27)	5.69 ^a^ (±0.04)	5.41 ^a^ (±0.10)	5.52 ^bc^ (±0.07)
	1000 mg/L	5.64 ^b^ (±0.35)	5.51 ^ab^ (±0.01)	5.55 ^a^ (±0.06)	5.56 ^b^ (±0.07)	5.75 ^d^ (±0.16)
GIX	200 mg/L	116.50 ^a^ (±18.07)	84.00 ^a^ (±35.05)	67.50 ^a^ (±4.93)	98.00 ^a^ (±38.34)	101.50 ^ab^ (±11.50)
	400 mg/L	79.00 ^b^ (±47.10)	67.00 ^a^ (±3.29)	73.50 ^a^ (±11.50)	127.00 ^a^ (±7.67)	84.50 ^a^ (±61.89)
	600 mg/L	133.50 ^a^ (±7.12)	127.00 ^b^ (±6.57)	81.00 ^a^ (±42.72)	125.50 ^a^ (±26.84)	120.50 ^b^ (±6.02)
	800 mg/L	71.00 ^b^ (±1.10)	24.50 ^c^ (±3.83)	64.50 ^a^ (±8.22)	66.50 ^b^ (±3.83)	92.00 ^ab^ (±7.67)
	1000 mg/L	88.50 ^b^ (±3.83)	92.50 ^a^ (±32.32)	81.00 ^a^ (±3.29)	109.50 ^a^ (±27.93)	85.50 ^a^ (±8.22)
VIX	200 mg/L	675.00 ^a^ (±123.52)	584.69 ^ab^ (±234.07)	318.98 ^a^ (±122.73)	741.12 ^a^ (±481.21)	765.92 ^a^ (±137.94)
	400 mg/L	588.98 ^ac^ (±540.57)	308.57 ^ac^ (±42.92)	331.53 ^a^ (±147.89)	1279.59 ^b^ (±34.88)	1042.55 ^a^ (±1097.35)
	600 mg/L	1100.20 ^b^ (±10.06)	918.37 ^b^ (±490.94)	719.18 ^b^ (±627.31)	1406.33 ^b^ (±612.78)	1065.82 ^a^ (±254.30)
	800 mg/L	320.92 ^c^ (±27.61)	16.94 ^c^ (±0.22)	330.41 ^a^ (±73.55)	302.76 ^a^ (±66.73)	796.73 ^b^ (±137.71)
	1000 mg/L	525.31 ^ac^ (±178.40)	744.49 ^ab^ (±618.37)	411.84 ^ab^ (±97.03)	1240.41 ^b^ (±443.99)	863.06 ^b^ (±236.30)

Values are averages of six replicates. Values in parenthesis represent ± standard deviations of means. Same and different superscripts on same column for a particular factor connote no significant difference and significant difference, respectively. K, L, M, N, and O represent *Serratia liquefaciens,* (OP830504), *S. liquefaciens* (OP830503), *Providencia rettgeri* (OP830491), *P. rettgeri* (OP830498), and *Bacillus cereus* (OP830501). FPG = final percent germination, MGT = mean germination time, GIX = germination index, and VIX = vigor index.

For the soybean seeds, significantly lowest final germination percentages were observed in treatment with metabolite concentrations of 200, 600, 800, and 1000 mg/L (metabolite from Isolate K), 800 mg/L (metabolite from Isolate M), and 600 mg/L (metabolite from Isolate N), and 1000 mg/L (metabolite from Isolate O). In the case of mean germination time, significantly lowest values were recorded in the seeds that were treated with 200, 400, and 800 mg/L (metabolite from Isolate K), 200, 400, 600, and 1000 mg/L (metabolite from Isolate L), 200, 600, and 1000 mg/L (metabolite from Isolate M), 200, 400, and 800 mg/L (metabolite from Isolate N), 200, 400, and 600 mg/L (metabolite from Isolate M). Generally, highest germination index values were observed in the seeds that were treated with 400 mg/L, 600 and 1000 mg/L, 400 and 800 mg/L, and 400 and 800 mg/L of metabolites from Isolates K, M, N, and O, respectively.

For the seeds treated with metabolites from Isolates K, M, N, and O, significantly highest seedling vigor index values were observed in the seeds treated with 400 mg/L, 600 mg/L, 400 mg/L, and 600 mg/L, respectively ( Table 6).

**Table 6.** Germinability of the soybean seeds at the different concentrations of the respective metabolites.

Parameter	Concentration	Isolate K	Isolate L	Isolate M	Isolate N	Isolate O
FPG	200 mg/L	42.86 ^ab^ (±15.65)	42.86 ^a^ (±31.30)	71.43 ^a^ (±15.65)	50.00 ^ac^ (±7.82)	50.00 ^a^ (±7.82)
	400 mg/L	50.00 ^a^ (±7.82)	42.86 ^a^ (±31.30)	64.29 ^a^ (±7.82)	50.00 ^ad^ (±7.82)	57.14 ^a^ (±15.65)
	600 mg/L	42.86 ^ab^ (±0.00)	42.86 ^a^ (±15.65)	85.71 ^b^ (±0.00)	14.29 ^b^ (±0.00)	85.71 ^b^ (±0.00)
	800 mg/L	35.71 ^b^ (±7.82)	35.71 ^a^ (±23.47)	42.86 ^c^ (±15.65)	57.14 ^a^ (±0.00)	71.43 ^c^ (±0.00)
	1000 mg/L	42.86 ^ab^ (±15.65)	42.86 ^a^ (±15.65)	85.71 ^d^ (±0.00)	42.86 ^cd^ (±15.65)	35.71 ^d^ (±7.82)
MGT	200 mg/L	5.43 ^a^ (±0.08)	5.50 ^a^ (±0.00)	5.52 ^ab^ (±0.00)	5.59 ^ab^ (±0.10)	5.39 ^a^ (±0.08)
	400 mg/L	5.41 ^a^ (±0.10)	5.59 ^ab^ (±0.10)	5.55 ^a^ (±0.10)	5.55 ^a^ (±0.06)	5.35 ^a^ (±0.04)
	600 mg/L	5.66 ^bc^ (±0.17)	5.59 ^ab^ (±0.10)	5.47 ^b^ (±0.00)	5.75 ^bcd^ (±0.27)	5.36 ^a^ (±0.05)
	800 mg/L	5.52 ^ab^ (±0.31)	5.93 ^b^ (±0.62)	5.55 ^a^ (±0.06)	5.61 ^ad^ (±0.00)	5.54 ^b^ (±0.05)
	1000 mg/L	5.73 ^c^ (±0.00)	5.68 ^ab^ (±0.24)	5.51 ^ab^ (±0.01)	5.81 ^c^ (±0.09)	5.59 ^b^ (±0.15)
GIX	200 mg/L	66.50 ^ab^ (±26.84)	63.00 ^a^ (±46.01)	101.00 ^a^ (±23.00)	68.00 ^a^ (±5.48)	77.50 ^a^ (±8.22)
	400 mg/L	77.00 ^b^ (±7.67)	57.00 ^a^ (±39.44)	89.50 ^a^ (±4.93)	70.50 ^ac^ (±8.22)	91.00 ^ac^ (±23.00)
	600 mg/L	57.00 ^a^ (±6.57)	57.50 ^a^ (±16.98)	127.00 ^b^ (±0.00)	18.00 ^b^ (±3.29)	136.50 ^b^ (±3.83)
	800 mg/L	48.50 ^a^ (±0.55)	50.50 ^a^ (±44.37)	60.00 ^c^ (±19.72)	78.00 ^c^ (±0.00)	102.00 ^c^ (±3.29)
	1000 mg/L	54.00 ^a^ (±19.72)	58.00 ^a^ (±29.58)	124.00 ^b^ (±2.19)	49.00 ^d^ (±14.24)	50.00 ^d^ (±15.34)
VIX	200 mg/L	237.35 ^a^ (±216.63)	153.98 ^a^ (±133.13)	847.55 ^ac^ (±398.16)	305.10 ^a^ (±53.88)	439.29 ^a^ (±98.25)
	400 mg/L	390.10 ^b^ (±115.47)	355.31 ^a^ (±351.88)	581.94 ^ad^ (±139.39)	183.27 ^b^ (±62.15)	450.61 ^a^ (±234.07)
	600 mg/L	146.94 ^a^ (±12.74)	238.17 ^a^ (±160.29)	2450.20 ^b^ (±1569.39)	25.31 ^c^ (±17.44)	1251.43 ^b^ (±29.51)
	800 mg/L	154.29 ^a^ (±32.19)	304.90 ^a^ (±317.90)	266.12 ^a^ (±202.99)	319.59 ^a^ (±68.41)	703.57 ^c^ (±124.63)
	1000 mg/L	149.18 ^a^ (±68.19)	280.82 ^a^ (±229.82)	1249.59 ^cd^ (±98.59)	62.45 ^c^ (±11.18)	180.82 ^d^ (±50.08)

Values are averages of six replicates. Values in parenthesis represent ± standard deviations of means. Same and different superscripts on same column for a particular factor connote no significant difference and significant difference, respectively. K, L, M, N, and O represent *Serratia liquefaciens,* (OP830504), *S. liquefaciens* (OP830503), *Providencia rettgeri* (OP830491), *P. rettgeri* (OP830498), and *Bacillus cereus* (OP830501). FPG = final percent germination, MGT = mean germination time, GIX = germination index, and VIX = vigor index.

In the case of the sesame seeds, remarkably high final germination (> 90 %) was observed at all steeping duration for all steeping durations. Significantly lowest mean germination time was recorded for seeds treated with 600 mg/L (metabolites from isolates K and L), 1000 mg/L (metabolites from isolates M and O) and 200, 400, and 600 mg/L (metabolite from isolate N). Generally, significantly highest germination index was observed in seeds treated with 600 mg/L of metabolite from isolate K, 600 mg/L, 800 mg/L, and 1000 mg/L of metabolites from isolate L, 1000 mg/L of metabolites from isolate M, 200-600 mg/L of metabolites from isolate N, and 1000 mg/L of metabolites from isolate O. For seedling vigor index, significantly highest values were observed at 400 and 600 mg/L for metabolite K, 600 and 800 mg/L for metabolite L, 200 and 1000 mg/L for metabolite M, 200 mg/L for metabolite N, and 1000 mg/L for metabolite O ( Table 7).

**Table 7.** Germinability of the sesame seeds at the different concentrations of the respective metabolites.

Parameter	Concentration	Isolate K	Isolate L	Isolate M	Isolate N	Isolate O
FPG	200 mg/L	96.43 ^ab^ (±3.91)	85.71 ^a^ (±0.00)	100.00 ^a^ (±0.00)	100.00 ^a^ (±0.00)	92.86 ^a^ (±7.82)
	400 mg/L	100.00 ^a^ (±0.00)	85.71 ^a^ (±0.00)	100.00 ^a^ (±0.00)	100.00 ^a^ (±0.00)	92.86 ^a^ (±7.82)
	600 mg/L	100.00 ^a^ (±0.00)	92.86 ^b^ (±7.82)	92.86 ^b^ (±7.82)	100.00 ^a^ (±0.00)	92.86 ^a^ (±7.82)
	800 mg/L	92.86 ^b^ (±7.82)	100.00 ^c^ (±0.00)	92.86 ^b^ (±7.82)	92.86 ^b^ (±7.82)	92.86 ^a^ (±7.82)
	1000 mg/L	92.86 ^b^ (±7.82)	100.00 ^c^ (±0.00)	100.00 ^a^ (±0.00)	100.00 ^a^ (±0.00)	100.00 ^a^ (±0.00)
MGT	200 mg/L	5.20 ^a^ (±0.00)	5.32 ^a^ (±0.13)	5.30 ^a^ (±0.04)	5.23 ^ac^ (±0.04)	5.21 ^ab^ (±0.02)
	400 mg/L	5.20 ^a^ (±0.08)	5.18 ^b^ (±0.03)	5.38 ^b^ (±0.04)	5.22 ^a^ (±0.05)	5.17 ^a^ (±0.11)
	600 mg/L	5.06 ^b^ (±0.07)	5.03 ^c^ (±0.03)	5.33 ^ab^ (±0.01)	5.22 ^a^ (±0.05)	5.21 ^ab^ (±0.06)
	800 mg/L	5.14 ^c^ (±0.01)	5.21 ^b^ (±0.09)	5.37 ^b^ (±0.06)	5.32 ^b^ (±0.10)	5.25 ^b^ (±0.02)
	1000 mg/L	5.17 ^ac^ (±0.03)	5.13 ^b^ (±0.00)	5.16 ^c^ (±0.04)	5.30 ^bc^ (±0.04)	5.13 ^c^ (±0.00)
GIX	200 mg/L	168.25 ^a^ (±7.39)	138.00 ^a^ (±10.95)	164.50 ^a^ (±3.83)	171.50 ^a^ (±3.83)	161.00 ^a^ (±15.34)
	400 mg/L	175.00 ^a^ (±7.67)	151.00 ^b^ (±3.29)	157.50 ^a^ (±3.83)	172.00 ^a^ (±4.38)	164.50 ^a^ (±3.83)
	600 mg/L	189.00 ^b^ (±7.67)	178.50 ^c^ (±11.50)	150.50 ^b^ (±11.50)	172.00 ^a^ (±4.38)	161.00 ^a^ (±7.67)
	800 mg/L	168.00 ^a^ (±15.34)	172.50 ^c^ (±10.41)	147.00 ^b^ (±7.67)	150.50 ^b^ (±3.83)	157.50 ^a^ (±11.50)
	1000 mg/L	164.50 ^a^ (±11.50)	182.00 ^c^ (±0.00)	178.50 ^d^ (±3.83)	164.50 ^c^ (±3.83)	182.00 ^b^ (±0.00)
VIX	200 mg/L	470.20 ^a^ (±54.55)	377.14 ^a^ (±59.02)	542.86 ^ad^ (±10.95)	529.29 ^a^ (±10.17)	437.96 ^a^ (±39.79)
	400 mg/L	603.57 ^b^ (±151.01)	380.20 ^a^ (±3.35)	458.57 ^bc^ (±104.85)	478.57 ^b^ (±28.17)	433.16 ^a^ (±37.22)
	600 mg/L	682.14 ^b^ (±66.51)	491.63 ^bc^ (±137.49)	450.82 ^c^ (±53.88)	445.71 ^b^ (±20.34)	455.51 ^a^ (±45.61)
	800 mg/L	470.00 ^a^ (±17.21)	409.29 ^ab^ (±44.60)	407.96 ^c^ (±25.71)	457.45 ^b^ (±35.66)	432.76 ^a^ (±22.02)
	1000 mg/L	437.86 ^a^ (±85.29)	567.86 ^c^ (±88.42)	525.00 ^d^ (±30.52)	450.71 ^b^ (±69.64)	524.29 ^b^ (±0.00)

Values are averages of six replicates. Values in parenthesis represent ± standard deviations of means. Same and different superscripts on same column for a particular factor connote no significant difference and significant difference, respectively. K, L, M, N, and O represent *Serratia liquefaciens,* (OP830504), *S. liquefaciens* (OP830503), *Providencia rettgeri* (OP830491), *P. rettgeri* (OP830498), and *Bacillus cereus* (OP830501). FPG = final percent germination, MGT = mean germination time, GIX = germination index, and VIX = vigor index.

Furthermore, the final germination percentage of the okra seeds showed significantly lowest values in setups that were treated with 800 mg/L of metabolites from Isolates K and N, 200, 800, and 1000 mg/L of metabolite from Isolate L, 800 and 1000 mg/L of metabolite from Isolate M, and 200 mg/L of metabolite from Isolate O. In the case of mean germination time, seeds treated with the metabolites from Isolates M and N showed no significance difference at the different concentrations, while treatment in 800 mg/L and 200, 400, and 1000 mg/L of the metabolites from Isolates K and O showed significantly highest values, respectively. With respect to germination index, significantly highest values were recorded in the seeds treated with 400, 600, and 1000 mg/L (metabolite from Isolate K), 600 mg/L (metabolite from Isolate L), 200-800 mg/L (metabolite from Isolate M), and 800 and 1000 mg/L (metabolites from Isolates N and O). For vigor index, the seeds treated in the metabolites at 400, 600, and 1000 mg/L (Metabolite K), 600 mg/L (Metabolite L), 200-600 mg/L (Metabolites M and N), and 800-1000 mg/L (Metabolite K) ( Table 8).

**Table 8.** Germinability of the okra seeds at the different concentrations of the respective metabolites.

Parameter	Concentration	Isolate K	Isolate L	Isolate M	Isolate N	Isolate O
FPG	200 mg/L	28.57 ^a^ (±0.00)	28.57 ^a^ (±0.00)	42.86 ^a^ (±31.30)	42.86 ^a^ (±15.65)	28.57 ^a^ (±0.00)
	400 mg/L	42.86 ^b^ (±15.65)	50.00 ^b^ (±7.82)	42.86 ^a^ (±0.00)	42.86 ^a^ (±15.65)	42.86 ^b^ (±0.00)
	600 mg/L	42.86 ^b^ (±15.65)	64.29 ^c^ (±7.82)	42.86 ^a^ (±15.65)	28.57 ^b^ (±0.00)	35.71 ^c^ (±7.82)
	800 mg/L	7.14 ^c^ (±7.82)	28.57 ^a^ (±0.00)	28.57 ^ab^ (±0.00)	7.14 ^c^ (±7.82)	64.29 ^d^ (±7.82)
	1000 mg/L	50.00 ^b^ (±7.82)	35.71 ^a^ (±7.82)	14.29 ^b^ (±0.00)	64.29 ^d^ (±7.82)	71.43 ^e^ (±0.00)
MGT	200 mg/L	5.37 ^a^ (±0.15)	5.37 ^a^ (±0.15)	5.44 ^a^ (±0.06)	5.26 ^a^ (±0.17)	5.50 ^a^ (±0.00)
	400 mg/L	5.37 ^a^ (±0.15)	5.43 ^ab^ (±0.08)	5.41 ^a^ (±0.10)	5.67 ^a^ (±0.06)	5.50 ^a^ (±0.00)
	600 mg/L	5.23 ^a^ (±0.00)	5.54 ^b^ (±0.05)	5.50 ^a^ (±0.00)	5.86 ^a^ (±0.15)	5.41 ^b^ (±0.10)
	800 mg/L	5.75 ^b^ (±3.01)	5.37 ^a^ (±0.15)	5.50 ^a^ (±0.00)	5.75a (±3.01)	5.32 ^c^ (±0.04)
	1000 mg/L	5.27 ^a^ (±0.05)	5.44 ^ab^ (±0.23)	5.50 ^a^ (±0.00)	5.50 ^a^ (±0.00)	5.47 ^ab^ (±0.09)
GIX	200 mg/L	45.50 ^a^ (±3.83)	45.50 ^a^ (±3.83)	66.50 ^a^ (±49.84)	74.00 ^a^ (±33.96)	42.00 ^a^ (±0.00)
	400 mg/L	66.50 ^b^ (±19.17)	77.00 ^b^ (±15.34)	66.50 ^a^ (±3.83)	57.00 ^a^ (±23.00)	63.00 ^b^ (±0.00)
	600 mg/L	73.50 ^b^ (±26.84)	91.50 ^c^ (±8.22)	63.00 ^a^ (±23.00)	33.00 ^b^ (±3.29)	56.00 ^b^ (±15.34)
	800 mg/L	10.50 ^c^ (±11.50)	45.50 ^a^ (±3.83)	42.00 ^ab^ (±0.00)	10.50 ^bc^ (±11.50)	105.00 ^c^ (±15.34)
	1000 mg/L	84.00 ^b^ (±15.34)	53.00 ^a^ (±4.38)	21.00 ^b^ (±0.00)	94.50 ^c^ (±11.50)	106.00 ^c^ (±6.57)
VIX	200 mg/L	83.06 ^a^ (±9.61)	106.94 ^a^ (±20.12)	285.10 ^a^ (±275.65)	217.96 ^a^ (±124.30)	84.08 ^a^ (±16.54)
	400 mg/L	181.02 ^b^ (±105.74)	201.94 ^b^ (±2.12)	253.16 ^ab^ (±34.54)	174.08 ^ab^ (±76.68)	196.53 ^a^ (±55.00)
	600 mg/L	212.24 ^b^ (±126.09)	410.41 ^c^ (±160.74)	205.51 ^ab^ (±109.32)	93.06 ^ab^ (±15.20)	205.71 ^a^ (±81.82)
	800 mg/L	10.82 ^a^ (±11.85)	66.73 ^a^ (±14.53)	99.18 ^bc^ (±21.01)	15.20 ^b^ (±16.66)	479.39 ^b^ (±223.78)
	1000 mg/L	232.04 ^b^ (±70.42)	126.43 ^ab^ (±45.94)	29.69 ^c^ (±7.49)	621.53 ^c^ (±274.87)	589.80 ^b^ (±40.24)

Values are averages of six replicates. Values in parenthesis represent ± standard deviations of means. Same and different superscripts on same column for a particular factor connote no significant difference and significant difference, respectively. K, L, M, N, and O represent *Serratia liquefaciens,* (OP830504), *S. liquefaciens* (OP830503), *Providencia rettgeri* (OP830491), *P. rettgeri* (OP830498), and *Bacillus cereus* (OP830501). FPG = final percent germination, MGT = mean germination time, GIX = germination index, and VIX = vigor index.

### Detected compounds in the metabolites

In the metabolite from *S. liquefaciens* AYO-O, the major compounds that were detected included methyl lactate (10.40%), 9,12-octadecadienoic acid (Z,Z)- (17.50%), n-hexadecanoic acid (13.38%), phytol (5.96%), oleic acid (11.48%) and 9,12-octadecadienoic acid (Z,Z)- (17.01%). Also, for the metabolite from *P. rettgeri* (OP830491), n-hexadecanoic acid (14.13%), octadecane (7.90%), phytol (9.31%), 11,14,17-eicosatrienoic acid, methyl ester (5.96%), lupeol (7.74%), stigmasterol (15.00%) and β-sitosterol (12.19%) were the most dominant compounds ( Table 9).

**Table 9.** Detected compounds in the metabolites.

Peak #	RT	Compound detected	Comp. wt%	RT	Compound detected	Comp. wt%
	**Metabolite from *S. liquefaciens* (OP830504)**			**Metabolite from *P. rettgeri* (OP830491)**		
1	2.50	Methyl lactate	10.40	2.54	Methyl lactate	2.28
2	2.99	Cyclohexanol, 5-methyl-2-(1-methylethyl)-	3.93	8.00	Cyclohexanol, 5-methyl-2-(1-methylethyl)-	5.00
3	4.46	Pentanoic acid, 2-methylbutyl ester	1.38	12.48	Pentanoic acid, 2-methylbutyl ester	2.51
4	8.50	9,12-Octadecadienoic acid (Z,Z)-	17.50	14.23	9,12-Octadecadienoic acid (Z,Z)-	2.07
5	9.52	Tetradecanoic acid	1.57	16.60	Tetradecanoic acid	2.55
6	15.15	Dibutyl phthalate	1.39	17.58	Dibutyl phthalate	1.97
7	16.00	9-Octadecenoic acid (Z)-, methyl ester	3.97	25.00	n-Hexadecanoic acid	14.13
8	21.00	3,7,11,15-tetramethyl-2-hexadecen-1-ol	1.29	30.00	3,7,11,15-tetramethyl-2-hexadecen-1-ol	1.52
9	25.00	Octadecane	2.03	31.97	Octadecane	7.90
10	32.00	n-Hexadecanoic acid	13.38	35.51	9-Octadecenoic acid (Z)-, methyl ester	1.69
11	34.50	Phytol	5.96	36.25	Phytol	9.31
12	35.81	Oleic acid	11.48	37.62	Oleic acid	2.98
13	37.52	9,12-Octadecadienoic acid (Z,Z)-	17.01	39.94	9,12-Octadecadienoic acid (Z,Z)-	1.25
14	39.00	11,14,17-Eicosatrienoic acid, methyl ester	3.89	41.00	11,14,17-Eicosatrienoic acid, methyl ester	5.96
15	41.98	Squalene	2.51	41.50	Lupeol	7.74
16	44.50	Stigmasterol	1.83	43.50	Stigmasterol	15.00
17				44.23	β-Sitosterol	12.19
18				44.99	Squalene	2.78
	**Metabolite from *S. liquefaciens* (OP830503)**			**Metabolite from *P. rettgeri* (OP830498),),**		
1	7.00	Methyl lactate	1.90	8.02	Methyl lactate	1.20
2	14.23	Cyclohexanol, 5-methyl-2-(1-methylethyl)-	1.86	10.50	Cyclohexanol, 5-methyl-2-(1-methylethyl)-	5.73
3	15.98	Pentanoic acid, 2-methylbutyl ester	1.94	11.43	Pentanoic acid, 2-methylbutyl ester	2.31
4	18.80	9,12-Octadecadienoic acid (Z,Z)-	4.90	13.25	9,12-Octadecadienoic acid (Z,Z)-	6.05
5	20.50	Tetradecanoic acid	8.85	16.50	Tetradecanoic acid	6.26
6	23.11	n-Hexadecanoic acid	2.92	20.81	Dibutyl phthalate	3.61
7	24.06	Dibutyl phthalate	1.55	23.03	9-Octadecenoic acid (Z)-, methyl ester	2.93
8	26.98	3,7,11,15-tetramethyl-2-hexadecen-1-ol	3.83	26.02	3,7,11,15-tetramethyl-2-hexadecen-1-ol	17.54
9	28.15	Octadecane	3.14	28.50	Octadecane	3.99
10	31.50	9-Octadecenoic acid (Z)-, methyl ester	1.90	32.50	n-Hexadecanoic acid	21.96
11	33.98	Phytol	1.96	35.71	Phytol	3.88
12	35.50	Oleic acid	29.23	37.20	Oleic acid	1.63
13	37.00	9,12-Octadecadienoic acid (Z,Z)-	2.62	37.80	9,12-Octadecadienoic acid (Z,Z)-	12.45
14	39.00	11,14,17-Eicosatrienoic acid, methyl ester	25.17	39.75	11,14,17-Eicosatrienoic acid, methyl ester	2.56
15	40.00	Squalene	4.37	40.75	Squalene	3.04
16	48.49	Stigmasterol	1.94	43.52	Stigmasterol	3.27
17	54.00	Lupeol	1.66	48.21	Stearyltrimethylammonium chloride	1.18
	**Metabolite from *Bacillus cereus* (OP830501)**					
1	7.61	Methyl lactate	4.06	2.03		
2	14.13	Cyclohexanol, 5-methyl-2-(1-methylethyl)-	2.13	1.73		
3	15.98	Pentanoic acid, 2-methylbutyl ester	2.67	1.87		
4	18.80	9,12-Octadecadienoic acid (Z,Z)-	4.27	5.83		
5	20.50	Tetradecanoic acid	15.00	16.24		
6	23.11	n-Hexadecanoic acid	3.20	2.51		
7	24.00	Dibutyl phthalate	4.03	2.55		
8	26.98	3,7,11,15-tetramethyl-2-hexadecen-1-ol	4.04	3.29		
9	28.15	Octadecane	2.16	1.82		
10	31.50	9-Octadecenoic acid (Z)-, methyl ester	1.07	1.80		
11	33.98	Phytol	1.25	1.52		
12	35.50	Oleic acid	26.69	27.33		
13	37.00	9,12-Octadecadienoic acid (Z,Z)-	5.03	4.10		
14	39.00	11,14,17-Eicosatrienoic acid, methyl ester	19.22	20.63		
15	40.00	Squalene	3.19	3.00		
16	48.49	Stigmasterol	1.06	1.81		
17	54.00	Lupeol	0.92	1.60		

In addition, the metabolites from *S. liquefaciens* AYO-N revealed the presence of tetradecanoic acid (8.85%), phytol (29.23%), and 11,14,17-eicosatrienoic acid, methyl ester (25.17%) as the most dominant species. In the case of the metabolite from *P. rettgeri* AYO-I, cyclohexanol, 5-methyl-2-(1-methylethyl)- (5.73%), 9,12-octadecadienoic acid (Z,Z)- (6.05%), tetradecanoic acid (6.26%), 3,7,11,15-tetramethyl-2-hexadecen-1-ol (17.54%), n-hexadecanoic acid (21.96%), and 9,12-octadecadienoic acid (Z,Z)- (12.45%) were the most dominant moieties ( Table 9).

Finally, in the metabolite from *B. cereus* AYO-L, tetradecanoic acid (15.00%), oleic acid (26.69%) and 11,14,17-eicosatrienoic acid, and methyl ester (19.22%) were the most dominant species ( Table 9).

## Discussion

Seed germination is fundamental to plant development and affects productivity, ^34^^,^^35^ and seeds that germinate vigorously produce better yields. ^36^ Generally, for the cowpea seeds, the final germination percentage reached a significantly highest value at shorter steeping durations (1, 2, or 3 h), then decreased afterward. However, for Metabolites N and O, all values were statistically the same or nearly so. With the soybean seeds, the final germination percentage increased with increasing steeping duration until 1, 2, or 3 h, then decreasing afterward, even though this decrease was sometimes insignificant. Although microbial metabolites can promote germination, ^37– 39^ the result for cowpea and soybean could be ascribed to nutrient and electrolyte leakage at prolonged steeping duration. ^40^ Long steeping durations can result in over-imbibition, ^41^ leading to swollen seeds that may not germinate. Microbial deterioration can occur in large endosperm seeds (soybean and cowpea) when primed for longer durations, especially when the seeds are sown immediately without drying, as in this study. The results of this study show that steeping soybean and cowpea for shorter periods is sufficient for maximal germination values. Through the application of the traditional priming technique that involves re-drying the seeds, higher optimal steeping durations of 6 hours (cowpea), 8 and 18 hours (soybean) were reported. ^42^^,^^43^^,^^44^ In this study, seeds were planted directly without re-drying.

Steeping duration did not seem to significantly impact the final germination pattern of the sesame seeds, as high values were distributed without order at the different steeping durations, and there was no significant difference between them. The sesame seeds did not become over-bloated as in cowpea and soybean. Sesame seeds are comparatively impervious and have a small endosperm. A high optimal steeping duration (12 hours) was recorded by Tizazu *et al*. ^45^ using the traditional priming method.

There was also no order to the distribution of FGP values for okra, indicating that the steeping duration did not significantly influence germination. This uneven germination pattern could be due to the hard seed coat of okra, ^46^ which perhaps limited imbibition. This hard seed coat likely resulted in high maximal steeping durations of 12 h ^47^ and 48 h ^48^ for okra. Insufficient imbibition can lead to germination delay, ^49^ a situation that could have happened with a hardy seed such as okra in this study since the highest steeping duration for this study was 5 hours, and it posted low final germination values.

The effect of steeping duration on mean germination time was negligible for all the crops, as there was no pattern in the distribution of values. All the values obtained “congregated’ around five (5) days. Hence, it does not make any agronomic sense to attach much importance to the significant results here. However, other researchers have reported better mean germination times in cucumber, ^50^ tomato seeds, ^51^ guava seeds, ^52^ cowpea seeds. ^53^

A high germination index shows that germinated seeds appeared faster. In the case of the cowpea seeds, there was a general decrease in values with increasing steeping time for all isolates, and this decline was significant for some metabolites used. There was also a general but insignificant decline from lower to higher steeping durations with the soybean seeds. However, steeping time did not significantly affect the germination index of the sesame and okra seeds. Okra required a 48-hour steeping duration for maximal germination index. ^48^ The highest germination index was observed when soybean seeds were treated for 12 hours in PEG solution. ^54^ Both studies utilized the traditional priming methods that involves seed re-drying.

The vigor index reached its statistical highest at a lower steeping duration in all the metabolites in the case of cowpea, then decreased afterward, and this same pattern was also obtained for the soybean and sesame seeds. Seeds can over-imbibe at longer steeping durations, impeding germination. This can result in reduced seedling vigor index values since final germination percentage is a computational component of seedling vigor index. Over-imbibition is a potential priming pitfall for seeds with large endosperms, such as cowpeas and soybean. Six (6) hours was the best priming duration for biomass production in soybean, ^55^ and 12 hours for the highest seedling vigor index in soybean by Sadeghi *et al*. ^54^

There was generally no observable pattern in FPG for cowpea, soybean, sesame, and okra seeds with concentration. Erratic drops in values were observed for the okra seeds. Okra has irregular germination pattern. ^56^ Similarly, germination was concentration-independent in the biopriming of canola seeds with varying concentrations of bacterial cell-free supernatants of *Devosia* sp. (SL43). ^57^ While increasing concentration did not impact germination negatively, it is usually not the case with chemical or hormonal priming where the impact of priming on germination and seedling parameters seems to always be concentration-dependent, with drastic effects at higher concentrations. ^58^^,^^59^

Metabolite concentration did not produce an effect of considerable agronomic proportion on mean germination time in all the crops, as values clustered around five days. Concerning the germination index, there was no clear pattern for cowpea and, generally, for the soybean and okra seeds. For the metabolites from isolates L, M, and O used in the priming of the sesame seeds, there was a rare increase in GIX with increasing concentration, which was also significant.

For the cowpea seeds, the seedling vigor index gradually peaked at lower concentration for all isolates, then decreased with increasing concentration, although the decreases were statistically insignificant at times. Similarly, the seedling vigor index peaked at a lower concentration, and then a significant decline was observed for the soybean seeds. Sesame recorded no clear pattern with increasing concentration; however, in the case of Isolate K, it peaked at a lower concentration, then decreased steadily afterward, and for Isolate L, it rose steadily and peaked significantly at the highest concentration. Mostly, there was no directional change in seedling vigor index with metabolite concentration for okra seeds treated in these metabolites. The hard seed coat of okra is responsible for the ambiguous response of okra to priming at different concentrations. The significantly highest value for okra was not limited to lower steeping periods, which occurred either 3 or 4 h for virtually all the metabolite treatments. A high steeping duration of 48 hours produced the best vigor index in okra. ^48^ The seed coat of okra limits ample imbibition at short steeping periods.

The GC-MS analysis of the extracts detected the presence of several metabolites in the metabolome of each of them, some of which were common to all isolates. The compounds detected belong to different classes, such as, alkanes, alcohols, carboxylic acids, esters, and terpenes. The ability of alcohols such as 2,3-butanediol produced by *Bacillus* spp to promote the growth of *Arabidopsis thaliana* has been reported. ^60^^–^^62^ Tetrahydrofuran-3-ol and 2-heptanone 2-ethyl-1-hexanol from *Bacillus* species can improve the growth of *A. thaliana* and tomato. ^63^

Oleic acid was detected in the metabolome of some rhizobacteria. ^64^ It was detected in all five strains in this study. n-hexadecanoic acid, a metabolite detected in the metabolome of two of the isolates in this study, and hexadecane can improve the growth of *Vigna radiata.* ^65^

## Conclusion

This study revealed the dynamic of metabolite priming at different steeping duration and metabolite concentration. The impact of steeping duration in metabolite priming was revealed in this study. It showed that seeds with large endosperms can become over-bloated at longer steeping durations, thereby impeding seed germination. Higher concentration was inhibitory to germination. Sesame and okra were the least affected seeds with regard to metabolite priming. However, the function of the various metabolites in the isolates sampled needs to be properly investigated to identify the bioactive metabolites responsible for growth promotion. The organisms themselves will have to be further studied to better understand the production of the bioactive metabolites.

## Data availability

Figshare. Raw data on germinability parameters at different metabolite concentrations and steeping duration in microbial metabolite. DOI: https://doi.org/10.6084/m9.figshare.23284865.v1. ^66^
