# Supplementary material for: Effects of priming duration and rhizosphere bacteria metabolite concentration on the germinability of cowpea, soybean, sesame, and okra seeds
Source: F1000Res. 2024 Oct 7;12:781. Originally published 2023 Jul 5. [Version 5] doi: 10.12688/f1000research.137322.5 (PMC11450368; doi:10.12688/f1000research.137322.5)
Supplement: Peer review history

# Reviewer report: Reviewer response for version 5

**DOI:** 10.5256/f1000research.172328.r329600

**Published:** 2024-10-17

**Authors:** Olatunde Samuel Dahunsi

**Affiliations:** 1 Microbiology Programme, College of Agriculture, Engineering and Science, Bowen University, Iwo, Osun, Nigeria

Authors have satisfactorily addressed all my previous concerns. I now approve this submission for acceptance and indexing.

Is the work clearly and accurately presented and does it cite the current literature?

Yes

If applicable, is the statistical analysis and its interpretation appropriate?

Yes

Are all the source data underlying the results available to ensure full reproducibility?

Yes

Is the study design appropriate and is the work technically sound?

Partly

Are the conclusions drawn adequately supported by the results?

Yes

Are sufficient details of methods and analysis provided to allow replication by others?

Yes

Reviewer Expertise:

NA

I confirm that I have read this submission and believe that I have an appropriate level of expertise to confirm that it is of an acceptable scientific standard.

# Reviewer report: Reviewer response for version 4

**DOI:** 10.5256/f1000research.169416.r306176

**Published:** 2024-10-03

**Authors:** Sowmyalakshmi Subramanian

**Affiliations:** 1 Department of Plant Sciences, MacDonald Campus, McGill University, Montreal, Québec, Canada

Thank you for making the changes.

Is the work clearly and accurately presented and does it cite the current literature?

No

If applicable, is the statistical analysis and its interpretation appropriate?

Partly

Are all the source data underlying the results available to ensure full reproducibility?

Yes

Is the study design appropriate and is the work technically sound?

Yes

Are the conclusions drawn adequately supported by the results?

Partly

Are sufficient details of methods and analysis provided to allow replication by others?

Yes

Reviewer Expertise:

NA

I confirm that I have read this submission and believe that I have an appropriate level of expertise to confirm that it is of an acceptable scientific standard.

# Reviewer report: Reviewer response for version 4

**DOI:** 10.5256/f1000research.169416.r325426

**Published:** 2024-09-20

**Authors:** Olatunde Samuel Dahunsi

**Affiliations:** 1 Microbiology Programme, College of Agriculture, Engineering and Science, Bowen University, Iwo, Osun, Nigeria

I have reviewed the manuscript appropriately. The study was well conceived, designed and executed while the presented results are commendable. I have no challenge reading through the manuscript and can recommend acceptance after some minor revision. My comments includes:

1. There is need to improve the English language usage throughout the manuscript
2. The keyword section is not acceptable in the current state all keywords are directly taken from the manuscript title besides that the number are few. I encourage authors to overhaul the section by selecting words or phrases that best describe the content of the manuscript and that can be used for indexing and abstracting purposes.
3. In the method section, details of bacterial isolation and characterization should be given as well as those of metabolite characterization.
4. The provided ascension numbers should be well clarified.
5. Generally, authors need to overhaul the grammar in this manuscript.

Is the work clearly and accurately presented and does it cite the current literature?

Yes

If applicable, is the statistical analysis and its interpretation appropriate?

Yes

Are all the source data underlying the results available to ensure full reproducibility?

Yes

Is the study design appropriate and is the work technically sound?

Partly

Are the conclusions drawn adequately supported by the results?

Yes

Are sufficient details of methods and analysis provided to allow replication by others?

Yes

Reviewer Expertise:

English usage, methods and general formatting

I confirm that I have read this submission and believe that I have an appropriate level of expertise to confirm that it is of an acceptable scientific standard, however I have significant reservations, as outlined above.

# Response

**Published:** 2024-09-27

**Authors:** Oghenerobor B. Akpor

**Affiliations:** Biological Sciences, Afe Babalola University, Ado Ekiti, Ekiti, Nigeria

**Dear Reviewer,**

 Please find within this document our responses to your comments.

**Comment:** There is need to improve the English language usage throughout the manuscript

**Response:** The manuscript has been further reviewed to improve presentation style.

**Comment:** The keyword section is not acceptable in the current state all keywords are directly taken from the manuscript title besides that the number are few. I encourage authors to overhaul the section by selecting words or phrases that best describe the content of the manuscript and that can be used for indexing and abstracting purposes.

**Response: **The necessary corrections have been made throughout the manuscript.

**Comment:** In the method section, details of bacterial isolation and characterization should be given as well as those of metabolite characterization.

**Response: **This section has been overhauled.

**Comment:** The provided ascension numbers should be well clarified.

**Response:** The necessary corrections have been made.

**Comment: **Generally, authors need to overhaul the grammar in this manuscript.

**Response: **The manuscript has been extensively reviewed to improve grammar and style.

 Thanks.

# Reviewer report: Reviewer response for version 3

**DOI:** 10.5256/f1000research.167213.r285910

**Published:** 2024-07-10

**Authors:** Sowmyalakshmi Subramanian

**Affiliations:** 1 Department of Plant Sciences, MacDonald Campus, McGill University, Montreal, Québec, Canada

Comment 5: Statistical analysis - 

 Authors' response _  We are of the opinion that the alphabet separation method of statistical presentation is adequate. The addition of *p*-values for pairwise comparisons will make the results somewhat ambiguous, almost incomprehensible, and unsightly because it will require the inclusion of hundreds of *p*-values. One table alone will generate 90 *p*-values.

 Review comment - p-value is of importance in any statistical analysis and it needs to be included in the Tables. If not in the main text, it certainly has to be in the supplementary tables. It is not about how unsightly the tables look, but that they have a meaning to the data analyzed.

Is the work clearly and accurately presented and does it cite the current literature?

No

If applicable, is the statistical analysis and its interpretation appropriate?

Partly

Are all the source data underlying the results available to ensure full reproducibility?

Yes

Is the study design appropriate and is the work technically sound?

Yes

Are the conclusions drawn adequately supported by the results?

Partly

Are sufficient details of methods and analysis provided to allow replication by others?

Yes

Reviewer Expertise:

Plant-Microbe Interactions, Genomics, Transcriptomics, Proteomics, Metabolomics, Biostimulants, Biocontrol

I confirm that I have read this submission and believe that I have an appropriate level of expertise to confirm that it is of an acceptable scientific standard, however I have significant reservations, as outlined above.

# Response

**Published:** 2024-07-19

**Authors:** Oghenerobor B. Akpor

**Affiliations:** Biological Sciences, Afe Babalola University, Ado Ekiti, Ekiti, Nigeria

**Dear Reviewer,**

 Thank you for the review, please find enclosed within this document our response to your comments.

**Comment: **p-value is of importance in any statistical analysis and it needs to be included in the Tables. If not in the main text, it certainly has to be in the supplementary tables. It is not about how unsightly the tables look, but that they have a meaning to the data analyzed.

**Response:** The p-vales have been included as tables in the supplementary files.

 Regards

# Reviewer report: Reviewer response for version 2

**DOI:** 10.5256/f1000research.160450.r274606

**Published:** 2024-05-23

**Authors:** Sowmyalakshmi Subramanian

**Affiliations:** 1 Department of Plant Sciences, MacDonald Campus, McGill University, Montreal, Québec, Canada

The title should be changed – Seed priming is the term used for soaking seeds in water or any other soaking treatment to enhance germination. Change all the “steeping/steep” words to “priming”.

 Also, the work is about the germination potential of metabolites extracted from cell-free supernatant of bacteria and not just from the bacteria. This way, components of the medium in which the culture was grown will also be present in this metabolite extract. The authors have to justify clearly that the compounds/metabolites affecting the germination are from the bacteria and not from the medium. What growth medium was used to grow these bacteria? And was the growth medium also tested as a control?

 If water was used as control, in this case there should be two controls – one with just water for germination and the other with water as a seed prime. In fact, water as a seed prime works really well. If a treatment works better than seed primed water control, then seed priming makes sense.

 Kindly correct the SI units and the formatting. For example, 50℃ should be 50 ℃, 1000 mg L ^-1^ etc. Be consistent with this type of formatting through out the text.

 The results section should have statistical analysis data associated with the germination data and with the p-values obtained by the analysis, to keep in accordance with a scientific publication. This part is lacking in this work, although Tables 1 (Cowpea), 2 (Soybean), 3 (Sesame) do represent alphabet separation but no p-values.

Is the work clearly and accurately presented and does it cite the current literature?

No

If applicable, is the statistical analysis and its interpretation appropriate?

Partly

Are all the source data underlying the results available to ensure full reproducibility?

Yes

Is the study design appropriate and is the work technically sound?

Yes

Are the conclusions drawn adequately supported by the results?

Partly

Are sufficient details of methods and analysis provided to allow replication by others?

Yes

Reviewer Expertise:

Plant-Microbe Interactions, Genomics, Transcriptomics, Proteomics, Metabolomics, Biostimulants, Biocontrol

I confirm that I have read this submission and believe that I have an appropriate level of expertise to confirm that it is of an acceptable scientific standard, however I have significant reservations, as outlined above.

# Response

**Published:** 2024-05-28

**Authors:** Oghenerobor B. Akpor

**Affiliations:** Biological Sciences, Afe Babalola University, Ado Ekiti, Ekiti, Nigeria

Dear Reviewer,

 The responses to review comments are as follows:

**1. Comment:** The title should be changed – Seed priming is the term used for soaking seeds in water or any other soaking treatment to enhance germination.

**Response:** The suggested correction has been made.

**2. Comment:** Change all the “steeping/steep” words to “priming”.

**Response: **The necessary corrections have been made throughout the manuscript.

**3. Comment:** Also, the work is about the germination potential of metabolites extracted from cell-free supernatant of bacteria and not just from the bacteria. This way, components of the medium in which the culture was grown will also be present in this metabolite extract. The authors have to justify clearly that the compounds/metabolites affecting the germination are from the bacteria and not from the medium. What growth medium was used to grow these bacteria? And was the growth medium also tested as a control? If water was used as control, in this case there should be two controls – one with just water for germination and the other with water as a seed prime. In fact, water as a seed prime works really well. If a treatment works better than seed primed water control, then seed priming makes sense.

**Response: **Nutrient broth was used to grow the organisms. During preliminary investigation (data not reported) involving 75 isolates, two (2) controls were used. The controls were water and sterile nutrient broth. Elimination was based on the ability of an isolate to produce significantly higher vigor index than those obtained through both water and broth priming. It was this selection criteria that produced this group of 15 isolates used in this study.

**4. Comment:** Kindly correct the SI units and the formatting. For example, 50℃ should be 50 ℃, 1000 mg L ^-1^ etc. Be consistent with this type of formatting through out the text.

**Response:** Adequate attention has been paid to this and necessary corrections made.

**5. Comment:** The results section should have statistical analysis data associated with the germination data and with the p-values obtained by the analysis, to keep in accordance with a scientific publication. This part is lacking in this work, although Tables 1 (Cowpea), 2 (Soybean), 3 (Sesame) do represent alphabet separation but no p-values.

**Response: **We are of the opinion that the alphabet separation method of statistical presentation is adequate. The addition of *p*-values for pairwise comparisons will make the results somewhat ambiguous, almost incomprehensible, and unsightly because it will require the inclusion of hundreds of *p*-values. One table alone will generate 90 *p*-values.

 Thanks.

# Reviewer report: Reviewer response for version 1

**DOI:** 10.5256/f1000research.150476.r201475

**Published:** 2023-10-06

**Authors:** Hillary Righini

**Affiliations:** 1 Department of Agricultural and Food Sciences, Alma Mater Studiorum, University of Bologna, Bologna, Italy

Abstract

 First, why did the authors choose bacteria agents of human-infection for extraction of metabolite?? Could they represent risk also in agriculture for the environment and for the workers(in a view for future application)?  This is an important issue that must be well argued and explained.

 The work consists of only a test on seed germination, so I think that materials and methods should be more and better explained.

 Then, the authors must pay attention to the language. The text is difficult to read and it does not flow well. In particular, the abstract is very difficult to read because of English language.

 A careful checking for spelling and grammatical is required throughout the text and often the sentences have no sense.

 Materials and methods lack of many important details.

 Here, I reported some suggestions, but MORE are needed.

 ABSTRACT

 ‘Vigorous germination and growth are linked to crop yield.’ This sentence should introduce better the scope of this work. In this way it doesn’t mean anything.

 ‘For concentration it was either a case of lower concentration being optimal or there was no detectable pattern with concentration.’ It is difficult to read and understand.

 Through out the manuscript, there are several sentence like this, very difficult to understand.

 I would like to underline that ‘a seed can be treated, not steeped’. So, seeds were treated by immersion/dipping/soaking for a period.

 MATERIALS AND METHODS

 ‘.. until when needed.’, it better ‘until use’.

 ‘The bacterial strains were isolated using the standard pour-plating procedure’, it needs a reference.

 ‘After isolation, distinct colonies were streaked on nutrient agar plates to obtain pure cultures,’. How was carried out the isolation? How did you identified the bacteria?

 It is not clear the temporal succession of the methodological steps.

 Have you isolated the bacteria? And then identified? Or they were already isolated, identified and stored in your university??

 ‘Prior to use, the respective seeds were subjected to viability tests.’ Change with ‘First, a test was carried out on seeds in order to assess their viability’.

 ‘Preliminary viability testing was carried out by soaking approximately 100 seeds from a lot in 200 mL of sterile distilled in a 400 mL beaker and allowed to stand for 2 min. ‘ I suggest to change with ‘Preliminary viability testing was carried out by soaking 100 seeds in 200 mL of sterile distilled in a beaker for 2 min’.

 ‘approximately’ is not a scientific term.

 ‘sterile distilled’ what? Water, compounds?

 It is only one ‘viability test’ that includes two steps: first step for excluding those seeds that floated, the second step for testing the germination percentage. This part should be explained better.

 ‘cotton wool’ was sterilized?

 Has the cotton been moistened? Which were the environmental conditions during the incubation for 7 days? Temperature (day/night), humidity.

 ‘known concentration’, which ones?

 ‘respective’ which ones?

 ‘under 1, 2, 3, 4, and 5 h’. Not correct.  The right way is ‘FOR 1,2,3,4 or 5 hours.’ (For example, seeds were treated by immersion for 4 hours).

 Each formula needs a reference.

 It is not clear how the authors have weight the compounds to obtain 200 mg/L, 400 mg/L, 600 mg/L, 800 mg/L and 1000 mg/L.

 In materials and methods, there is no a statical paragraph. It is not clear how many repetitions. Where is the control?

 K, L, M, N, O: it is not clear their meaning. Moreover, in the results sometimes 'K, L, M, N, O' are reporte, sometimes the name of bacterial species. It is a confused way.

 ‘The bacterial strains were isolated using the standard pour-plating procedure’ explain the procedure and insert a reference for it.’

 ‘from rhizospheres within Afe Babalola University environment’: rhizosphere of which plant? How was this environment managed? Was there any crop, are still any crops?  Is it a resting soil?

 ‘Afe Babalola University’ change with ‘Afe Babalola University (Ekiti, Nigeria)’

 ‘All the seeds were sourced from local markets in Ado Ekiti, Ekiti State, Nigeria’. You must indicate the producer, the city, an the production and purchase date.

Is the work clearly and accurately presented and does it cite the current literature?

No

If applicable, is the statistical analysis and its interpretation appropriate?

No

Are all the source data underlying the results available to ensure full reproducibility?

No

Is the study design appropriate and is the work technically sound?

No

Are the conclusions drawn adequately supported by the results?

No

Are sufficient details of methods and analysis provided to allow replication by others?

No

Reviewer Expertise:

Agriculture

I confirm that I have read this submission and believe that I have an appropriate level of expertise to state that I do not consider it to be of an acceptable scientific standard, for reasons outlined above.

# Response

**Published:** 2023-12-20

**Authors:** Oghenerobor B. Akpor

**Affiliations:** Biological Sciences, Afe Babalola University, Ado Ekiti, Ekiti, Nigeria

**Dear Reviewer,**

**Please find enclosed our responses to your comments.**

**First, why did the authors choose bacteria agents of human-infection for extraction of metabolite?? Could they represent risk also in agriculture for the environment and for the workers (in a view for future application)?  This is an important issue that must be well argued and explained.**

**Answer:** *Serratia* is a genus of opportunistic pathogens of immunocompromised patients. The main pathogen in this genus is Serratia *marcescens* and *Serratia* *liquefaciens* are rarely implicated in infectious diseases. *Providencia* *rettgeri* is also a rare pathogen of humans and *Bacillus* *cereus* is a minor pathogen of foodborne illnesses. Moreover, strains of these organisms are common constituents of commercial preparations for plant growth promotion. This informed our decision to include them in this study. Nevertheless, the investigation of their pathogenicity will be carried out down the line in view of possible future application. Only then can their biosafety be ascertained.

**The work consists of only a test on seed germination, so I think that materials and methods should be more and better explained.**

**Answer: **The authors have gone to great lengths to expound the Material and Methods section.

**Then, the authors must pay attention to the language. The text is difficult to read and it does not flow well. In particular, the abstract is very difficult to read because of English language.**

**Answer: **The errors have been corrected. Significant improvements have been made to the composition and flow of the text.

**A careful checking for spelling and grammatical is required throughout the text and often the sentences have no sense.**

**Answer: **The authors have gone to great lengths to improve the grammar, style, and spelling in the text

**Materials and methods lack of many important details.**

**Answer: **Significant and important changes have been made.

**Here, I reported some suggestions, but MORE are needed.**

**ABSTRACT**

 ‘Vigorous germination and growth are linked to crop yield.’ This sentence should introduce better the scope of this work. In this way it doesn’t mean anything.

**Answer:** Rectified.

**‘For concentration it was either a case of lower concentration being optimal or there was no detectable pattern with concentration.’ It is difficult to read and understand.**

**Through out the manuscript, there are several sentences like this, very difficult to understand.**

**Answer: **Corrected.

**I would like to underline that ‘a seed can be treated, not steeped’. So, seeds were treated by immersion/dipping/soaking for a period.**

**Answer:** The necessary has been made.

**MATERIALS AND METHODS**

**‘.. until when needed.’, it better ‘until use’.**

**Answer:** Corrected.

 ‘The bacterial strains were isolated using the standard pour-plating procedure’, it needs a reference.

**Answer:** Corrected.

**‘After isolation, distinct colonies were streaked on nutrient agar plates to obtain pure cultures,’. How was carried out the isolation? How did you identified the bacteria?**

**Answer:** The requisite changes have been made.

**It is not clear the temporal succession of the methodological steps.**

**Answer:** Changes have been made. It now follows a logical succession.

**Have you isolated the bacteria? And then identified? Or they were already isolated, identified and stored in your university?**

**Answer: **They were isolated from a previous experiment separate from this study and stored on agar slants. They were identified through 16S rRNA.

**‘Prior to use, the respective seeds were subjected to viability tests.’ Change with ‘First, a test was carried out on seeds in order to assess their viability’.**

**Answer: **The suggested correction has been made

 ‘Preliminary viability testing was carried out by soaking approximately 100 seeds from a lot in 200 mL of sterile distilled in a 400 mL beaker and allowed to stand for 2 min. ‘ I suggest to change with ‘Preliminary viability testing was carried out by soaking 100 seeds in 200 mL of sterile distilled in a beaker for 2 min’.

**Answer: **The suggested correction has been made

 ‘ **approximately’ is not a scientific term.**

**Answer: **The suggested correction has been made

**‘sterile distilled’ what? Water, compounds?**

**Answer: Water. **The necessary correction has been made

 It is only one ‘viability test’ that includes two steps: first step for excluding those seeds that floated, the second step for testing the germination percentage. This part should be explained better.

**Answer: **The suggested correction has been made

 ‘cotton wool’ was sterilized?

**Answer: No.**

 Has the cotton been moistened? Which were the environmental conditions during the incubation for 7 days? Temperature (day/night), humidity.

**Answer: **Those were not measured.

**‘known concentration’, which ones?**

**Answer: **This has been corrected

**‘respective’ which ones?**

**Answer: **The 4 different seeds. It has been recast

**‘under 1, 2, 3, 4, and 5 h’. Not correct.  The right way is ‘FOR 1,2,3,4 or 5 hours.’ (For example, seeds were treated by immersion for 4 hours).**

**Answer: **The suggested correction has been made

**Each formula needs a reference.**

**Answer: **They were actually included in the original manuscript submitted.

**It is not clear how the authors have weight the compounds to obtain 200 mg/L, 400 mg/L, 600 mg/L, 800 mg/L and 1000 mg/L.**

**Answer: **Corrections made **.**

**In materials and methods, there is no a statical paragraph. It is not clear how many repetitions.**

**Answer: **The suggested correction has been made.

**Where is the control?**

**Answer: **No control because water is water, no concentration gradient.

 K, L, M, N, O: it is not clear their meaning. Moreover, in the results sometimes 'K, L, M, N, O' are reported, sometimes the name of bacterial species. It is a confused way.

**Answer: **The full names were only used in the presentation of the results of metabolite characterization to ensure brevity, elsewhere in the Result section the letters were used.

**‘The bacterial strains were isolated using the standard pour-plating procedure’ explain the procedure and insert a reference for it.’**

**Answer: **Rectified

 ‘from rhizospheres within Afe Babalola University environment’: rhizosphere of which plant? How was this environment managed? Was there any crop, are still any crops?  Is it a resting soil?

**Answer: **1. It’s a resting soil with various grasses. 2. The rhizospheres were not noted. 3. It was a managed grassland.

**‘Afe Babalola University’ change with ‘Afe Babalola University (Ekiti, Nigeria)’**

**Answer: **The suggested correction has been made

 ‘All the seeds were sourced from local markets in Ado Ekiti, Ekiti State, Nigeria’. You must indicate the producer, the city, an the production and purchase date.

**Answer: **Unfortunately, the seeds were not labelled. But they were procured from a seed dealer who supplies farmers with fresh seeds. Nonetheless, in order to ascertain their viability, the germination test was carried out

# Reviewer report: Reviewer response for version 1

**DOI:** 10.5256/f1000research.150476.r193085

**Published:** 2023-09-04

**Authors:** Debasis Mitra

**Affiliations:** 1 Microbiology, Crop Production Division, International Rice Research Institute, Manila, Metro Manila, Philippines; 2 Department of Microbiology, Raiganj University, Raiganj, West Bengal, India

The manuscript "Effects of steeping duration and concentration of metabolites from rhizosphere bacteria on germinability of cowpea ( *Vigna unguiculata*), soybean ( *Glycine max*), sesame ( *Sesamum indicum*) and okra ( *Abelmoschus esculentus*)" investigated a compelling topic to evaluate the impact of steeping duration and metabolite concentration on seed priming of five distinct crops, utilizing the metabolites of five bacterial isolates that were also characterized via Gas Chromatography-Mass Spectrometry (GC-MS). The results indicated that longer steeping duration may have a negative effect on larger endosperm seeds, such as cowpea and soybean, and further research is needed to isolate and purify the active compounds for additional studies and practical applications. Despite the fact that this study has many positive aspects and is suitable for this journal's scope, however, the manuscript needs to be revised critically and some important details need to be addressed.

 Minor Comments:

 Correct all grammatical mistakes. Verify that every citation in the text is linked to an appropriate reference in the reference section. Ensure that every reference has a textual citation that corresponds to it.

 Major Comments:

- Revise the title, remove the crops scientific name from the title, and add all in abstract.
- The abstract is a too general statement that does not convey the objective of your study and highlights the major flaws in your discussion of findings.
- Add Gas Chromatography-Mass Spectrometry (GC-MS) in Keywords
- “The bacterial strains were isolated from rhizospheres within Afe Babalola University environment.” rewrite it and give proper isolation and identification details.
- Metabolite extraction protocol must be explained in detail alone with GC-MS procedure.
- “Ethical approval” put it in end.
- NCBI accession numbers show in one place only, no need to write in every place. It's recommended to use it with the strain name or code.
  Example. OP830504 - *Serratia liquefaciens* AYO-O or AYO-O; OP830503 - *Serratia liquefaciens* AYO-N or AYO-N; OP830491 - *Providencia rettgeri* AYO-B /AYO-B....
- The conclusion is poorly written and lacks properly investigated findings and discussion regarding the role of metabolites in crop growth and development.

Is the work clearly and accurately presented and does it cite the current literature?

No

If applicable, is the statistical analysis and its interpretation appropriate?

I cannot comment. A qualified statistician is required.

Are all the source data underlying the results available to ensure full reproducibility?

Yes

Is the study design appropriate and is the work technically sound?

Yes

Are the conclusions drawn adequately supported by the results?

No

Are sufficient details of methods and analysis provided to allow replication by others?

Partly

Reviewer Expertise:

Plant Microbe Interactions, Soil Microbiology,Arbuscular Mycorrhizal Fungi, Biocontrol, Environmental Microbiology, Strigolactone

I confirm that I have read this submission and believe that I have an appropriate level of expertise to confirm that it is of an acceptable scientific standard, however I have significant reservations, as outlined above.

# Response

**Published:** 2023-12-20

**Authors:** Oghenerobor B. Akpor

**Affiliations:** Biological Sciences, Afe Babalola University, Ado Ekiti, Ekiti, Nigeria

Dear Reviewer,

 Please find enclosed within this document our response to your comments.

**Minor comments:**

 Correct all grammatical mistakes. Verify that every citation in the text is linked to an appropriate reference in the reference section. Ensure that every reference has a textual citation that corresponds to it.

**Response:** Adequate attention has been paid to the grammar, citation, and referencing.

**Major Comments:**

- Revise the title, remove the crops scientific name from the title, and add all in abstract.

**Response: **This has been attended to.

- The abstract is a too general statement that does not convey the objective of your study and highlights the major flaws in your discussion of findings.

**Response: **Rectified

- Add Gas Chromatography-Mass Spectrometry (GC-MS) in Keywords

**Response: **Rectified

- “The bacterial strains were isolated from rhizospheres within Afe Babalola University environment.” rewrite it and give proper isolation and identification details.

**Response: **Corrected

- Metabolite extraction protocol must be explained in detail alone with GC-MS procedure.

**Response: **Revised

- “Ethical approval” put it in end.

**Response: **The stipulated correction has been made

- NCBI accession numbers show in one place only, no need to write in every place. It's recommended to use it with the strain name or code.
  Example. OP830504 - *Serratia liquefaciens* AYO-O or AYO-O; OP830503 - *Serratia liquefaciens* AYO-N or AYO-N; OP830491 - *Providencia rettgeri* AYO-B /AYO-B....

**Response: Corrections have been made**

- The conclusion is poorly written and lacks properly investigated findings and discussion regarding the role of metabolites in crop growth and development.

**Response: **It has been reworked
